# Perspectives and Limitations of Tartaric Acid Diamides as Phase Change Materials for Sustainable Heat Applications

**DOI:** 10.1002/cssc.202500145

**Published:** 2025-04-16

**Authors:** Magdalena Gwóźdź, Natalia Siodłak, Anna Chrobok, Karolina Matuszek, Alina Brzęczek‐Szafran

**Affiliations:** ^1^ Department of Organic Chemical Technology and Petrochemistry Faculty of Chemistry Silesian University of Technology 44‐100 Gliwice Poland; ^2^ Chemistry Students Research Society Faculty of Chemistry Silesian University of Technology 44‐100 Gliwice Poland; ^3^ School of Chemistry Monash University Melbourne 3800 Victoria Australia

**Keywords:** H‐bonding interactions, phase change material, tartaric acid, thermal energy storage, thermal properties

## Abstract

Phase change materials (PCMs) with melting temperatures in the intermediate range (100–220 °C) have recently been in high demand for applications in solar and wind renewable energy storage. Such materials can help advance thermal battery technologies, e.g. Carnot batteries, that can reduce the amount of fossil fuels used to generate electricity, contributing to substantial savings in CO_2_ emissions. Recently, polyol esters have been recognized as robust PCMs with high stability and high energy storage density (up to 221 J g^−1^), additionally meeting sustainability and circularity criteria, being sourced from inexpensive, biorenewable tartaric acid (TA), which provides H‐bonding, boosting the esters’ thermal properties. However, the melting points of TA esters, which are below 100 °C, limit their suitability for applications in the intermediate temperature range. In this study, TA diamides are explored as candidates for thermal energy storage with improved melting temperatures ranging from 130 to 190 °C and melting enthalpies up to 173 J g^−1^. With the aid of differential scanning calorimetry (DSC), thermogravimetric analysis (TGA), and variable‐temperature Fourier‐transform infrared spectroscopy (FT‐IR), various perspectives and limitations of designing TA‐derived PCMs for sustainable heat use above 100 °C are investigated.

## Graphical abstract

In this study, tartaric acid diamides are explored for thermal energy storage with melting temperatures ranging from 130 to 190 °C and melting enthalpies reaching 173 J g^−1^. With the aid of differential scanning calorimetry, thermogravimetric analysis, and variable‐temperature infrared spectroscopy, various perspectives and limitations of designing tartaric acid‐derived phase change materials for sustainable heat use above 100 °C are investigated.

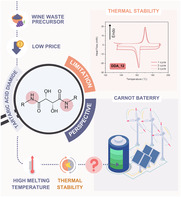

## 1 Introduction

Growing awareness of ongoing climate change, and the energy crisis has highlighted the importance of developing and using renewable energy technologies that offer low‐emission solutions to mitigate climate change and increase energy security, paving the way to a cleaner and more reliable energy future. This direction is supported by the global predictions stating that up to 2050 the electricity produced from fossil fuels will be reduced to only 21%, with solar energy responsible for 56% of production (62% of electricity was produced from fossil fuels in 2020).^[^ ^1^ ^]^ With increasing utilization of renewable energy, the challenge of maintaining consistent energy supplies, regardless of time or season, has gained prominence. In this context, sustainable thermal energy storage (TES), with a special emphasis on phase change materials (PCMs) that undergo a reversible transition between two states of matter, absorbing or releasing latent heat, has gained a significant level of interest. The most common are solid‐liquid PCMs, and their properties strictly depend on the melting point (*T*_m_) and enthalpy of fusion (Δ*H*_f_).^[^ ^2^ ^]^ PCMs can be integrated in various applications utilizing renewable energy, such as solar process heat, solar sorption cooling, or Carnot batteries, which combine heat storage within the PCM with a reversible heat pump/Carnot engine.^[^ ^3^, ^4^ ^]^ These technologies have potential to change the renewable energy storage market with their low cost, easy scalability, and being rare earth metal‐free. The primary requirement for a PCM in these applications is a specific melting point coupled with a high heat of fusion. The ideal temperature for optimization is between 100 and 220 °C.^[^ ^4^ ^]^

**Figure 1:**
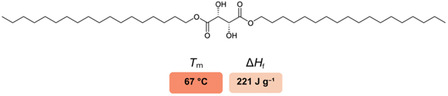
Examples of biobased TA esters as promising PCMs for applications with *T*_m_ < 100 °C.

To support green transformation, not only should technologies address aspects of sustainability but also the materials they rely on should do so (i.e., the PCM production process should minimize the environmental impact, they should eliminate resource scarcity and integrate well into the value chain of a circular economy). In this context, materials sourced from natural precursors with their accessibility, typically low toxicity, and recyclability can play a key role in advancing the concept of a circular economy. Recently, we demonstrated fully biobased tartaric acid (TA) esters as promising PCMs with remarkable thermal properties (Δ*H*_f_ values up to 221 J g^−1^ and stability for over 500 cycles) (**Figure** **1**).^[^ ^5^ ^]^ Nevertheless, these renewable fatty acid esters show *T*_m_ < 100 °C, which makes them attractive candidates for hot water applications and space heating but constrains their integration with renewable energy sources operating at higher temperatures (100–220 °C).^[^ ^6^ ^]^

Among organic materials, amides^[^ ^7^, ^8^, ^9^ ^]^ have emerged as promising TES candidates for renewable energy applications, with melting points in the desired temperature range (100–220 °C) and high latent heat values.^[^ ^2^, ^6^, ^10^ ^]^ The structure of diamides, especially the distance between functional groups and the length of the alkyl chain, have been widely discussed in the literature and highlighted as crucial factors dictating thermal properties.^[^ ^8^, ^9^ ^]^ A series of linear diamides with a formula of H_2_NCO–(CH_2_)_(*n*−2)_–CONH_2_ showed high *T*_m_ values up to 226 °C, depending on the number of methylene units in the system.^[^ ^11^ ^]^ For systems with an even number of methylene units, the *T*_m_ decreased from 226 °C for adipamide to 196 °C for tetradecanediamide, while for systems with an odd number of methylene units, the *T*_m_ values remained unchanged (≈177 °C). The investigated diamides showed high Δ*H*_f_ values up to 302 J g^−1^ for tetradecanediamide (the diamide with the longest alkyl chain). Again, the Δ*H*_f_ and Δ*S*_f_ values were influenced by the number of methylene groups in the system, favoring ones with an even number of carbon atoms compared to those with an odd number. The differences were attributed to the packing of the molecules in the solid state.

Another example of diamides with thermal properties suitable for applications in the intermediate temperature range is a group of diamides derived from fatty acids and diamines. The enhanced thermal stability of the diamide was attributed to the increased van der Waals interactions between the long alkyl chains and its high molecular weight, both of which significantly impact the degradation mechanism.^[^ ^12^ ^]^ This group of diamides was first reported in 2010 by Canik and Alkan,^[^ ^13^ ^]^ who reported hexamethylene dilauroyl amide and hexamethylene dipalmitoyl amide with *T*_m_ of 38.8 and 59.3 °C, respectively, and Δ*H*_f_ of 110 and 145 J g^−1^, respectively. The amides described were stable for up to 1000 heating and cooling cycles. On being further examined by Floros et al.^[^ ^14^ ^]^ the diamides gave higher *T*_m_ values of around 150 °C and higher latent heats exceeding 190 and 210 J g^−1^. The differences in the reported values were attributed to contamination, underlining the importance of the purification of the organic compounds to achieve the desired purity level for TES applications. Diamides with an even number of carbons (C_2_–C_10_) exhibited high *T*_m_ values, ranging from 159 °C for *N*, *N’*‐(ethane‐1,2‐diyl) didodecanamide to 141 °C for *N*, *N’*‐(decane‐1,10‐diyl) dioctadecanamide, and high Δ*H*_f_ values, reaching 250 J g^−1^ for *N*, *N’*‐(ethane‐1,2‐diyl) dioctadecanamide.^[^ ^8^, ^9^ ^]^ The high thermal stability of *N*, *N’*‐(decane‐1,10‐diyl) dioctadecanamide, which decomposed at 340 °C, additionally provided high operational stability.

Overall, the higher phase transition temperature of amides compared to esters can be ascribed to the stronger hydrogen bonds observed for the amides.^[^ ^15^ ^]^ As we have demonstrated for other various groups of organic compounds (organic salts,^[^ ^16^ ^]^ sugar acid esters,^[^ ^5^ ^]^ and urethanes^[^ ^17^ ^]^), H‐bonding interactions also have a positive impact on the Δ*H*_f_ values. Incorporation of renewable hydroxyl group‐rich TA moieties capable of forming H‐bonds into the structure of fatty acid esters allowed both the melting temperature and Δ*H*_f_ values to be increased compared to similar compound without hydroxyl groups, additionally supporting circularity and sustainability criteria.

Inspired by the extraordinary thermal properties and sustainability of TA esters, we aimed to develop analogous diamides, with melting temperatures in the intermediate range (100–220 °C), suitable for renewable solar/wind energy applications. This study focused on understanding the thermal behavior of TA diamides, with particular emphasis on their structural features, such as asymmetric configurations that influence hydrogen bonding interactions, the length of the alkyl chain, which affects van der Waals interactions, and combinations of these factors. Various perspectives and limitations of using inexpensive, renewable TA for the construction of PCMs that melt above 100 °C are elucidated and discussed in relation to diamides without the additional ‐OH groups. Our study aimed to establish structure−property relationships, which we believe are crucial for guiding the future design of sustainable PCMs.

## 2 Results and Discussion

### 2.1 The Isomers of TA Diamides

**Figure 2:**
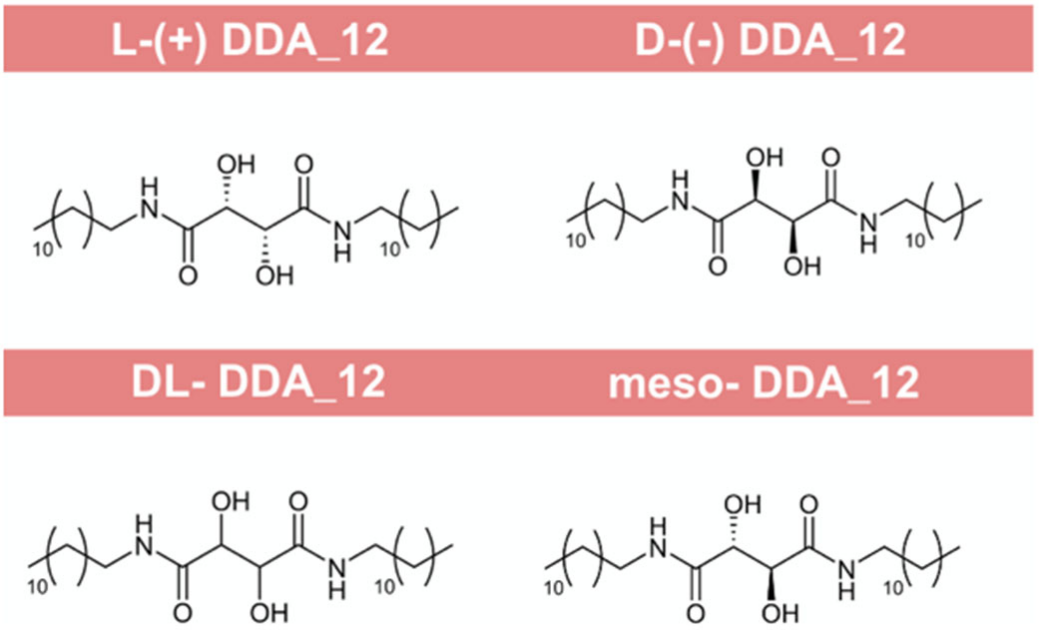
Structures of C_12_ amides derived from L‐(+)‐TA, D‐(−)‐TA, racemic TA mixture, and meso‐TA.

TA exists as a pair of enantiomers (L‐(+)‐TA and D‐(−)‐TA) and an achiral meso compound. As has been demonstrated for sugar alcohols, the geometric configuration and resulting H‐bonding strongly influences the thermal storage density of the isomers.^[^ ^18^, ^19^, ^20^ ^]^ Mannitol, for instance, has a high melting enthalpy of 308.3 J g^−1^, while the value for its isomer sorbitol is only 164.8 J g^−^^1^. To understand the influence of the ‐OH groups’ positions on the molecule's thermal properties, the amides of L‐(+)‐TA, D‐(−)‐TA, their racemic mixture, and the meso diastereomer were synthesized using dodecylamine (**Figure** **2**); these diamides are referred to as DDA_12. All synthesized amides had purity of >99% determined by high resolution mass spectrometry and nuclear magnetic resonance: ^1^HNMR and ^13^CNMR (Supporting Information).

Their thermal properties, including melting temperature (*T*_m_), crystallization temperature (*T*_*c*_), enthalpy of fusion (Δ*H*_f_), and crystallization enthalpy (Δ*H*_*c*_), were determined using differential scanning calorimetry (DSC). Thermal stability was investigated with thermogravimetric analysis (TGA), recording the decomposition temperature (*T*_d_) (Figure 8). Both the melting points and melting enthalpies of the isomers and their mixture varied significantly (**Table** **1**).

**Table 1:** Thermal properties of C_12_ amides derived from L‐(+)‐TA, D‐(−)‐TA, racemic TA mixture, and meso‐TA.

Compound	*T*_m_ [°C] ±2 °C	Δ*H*_f_ [J g^−1^] ±5%	*T*_c_ [°C] ±2 °C	Δ*H*_c_ [J g^−1^] ±5%	Δ*S*_f_ [J mol^−1^ K^−1^]
L‐(+)‐DDA_12	171	142 ± 7	162	143 ± 7	155
D‐(‐)‐DDA_12	161	115 ± 6	162	118 ± 6	127
DL‐DDA_12	62	62 ± 3	82	60 ± 3	83
	151	131 ± 7	149	127 ± 6	149
Meso‐DDA_12	130	132 ± 7	127	113 ± 6	156

The L‐(+)‐DDA_12 isomer had the highest melting point at 171 °C ± 2 °C and a melting enthalpy of 142 ± 7 J g^−1^, while the D‐(+)‐DDA_12 isomer showed a lower melting point of 161 °C ± 2 °C and the lowest melting enthalpy at 115 ± 6 J g^−1^. For their racemic mixture, two distinct melting peaks at 62 and 151 °C ± 2 °C were determined, along with two crystallization peaks at 82 and 149 °C ± 2 °C. Racemic mixtures frequently display a range of crystallization patterns, typically forming a conglomerate or a racemic compound,^[^ ^21^ ^]^ explaining the two distinct melting and crystallization peaks observed. The meso‐DDA_12 derivative had the lowest melting point of 130 °C ± 2 °C and a melting enthalpy of 132 ± 7 J g^−1^. Similar characteristics for the *T*_m_ values were observed for pure TA isomers, with the lowest *T*_m_ being that of the meso isomer.

**Figure 3:**
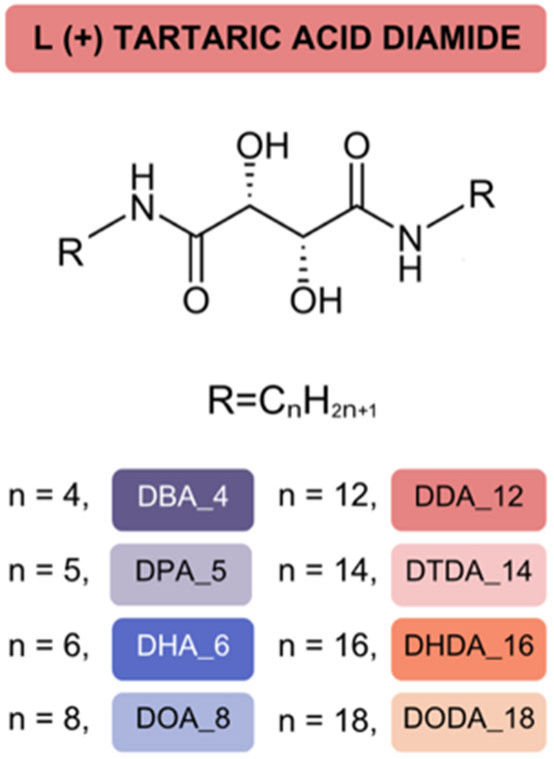
L‐(+)‐TA diamide series.

Based on the highest Δ*H*_f_ determined for the L‐(+)‐DDA_12 isomer, a series of diamides derived from L‐(+)‐TA, varying in alkyl chain length (4, 5, 6, 8, 12, 14, 16, and 18 carbons), were synthesized (**Figure** **3**) using a straightforward two‐step procedure. In the first step, 1,4‐dimethyl tartrate was produced through the esterification of TA with methanol,^[^ ^22^ ^]^ followed by condensation of the resulting 1,4‐dimethyl tartrate with an excess of the corresponding aliphatic amine in the presence of methanol or THF as the solvent (Scheme S1, Supporting Information). The reactions were performed over 72 h at temperatures ranging from 25 to 60 °C, depending on the amine used. The hydroxyl‐group‐rich amides were purified by crystallization from hot ethanol, followed by chloroform, and were subjected to DSC analysis over three heating and cooling cycles (Figure S26, Supporting Information). Their thermophysical properties are summarized in **Table** **2**.

**Table 2:** Thermal properties of TA diamides and their precursor, L‐(+)‐TA.

Compound	*T*_m_ [°C] ±2 °C	Δ*H*_f_ [J g^−1^] ±5%	*T*_c_ [°C] ±2 °C	Δ*H*_c_ [J g^−1^] ±5%	Δ*S*_f_ [J mol^−1^ K^−1^]	*T*_d_ [°C]
L‐(+)‐TA	Da)	–	–	–	–	172
L‐(+)‐DBA_4	190	160 ± 8	183	58 ± 3	88	266
L‐(+)‐DPA_5	189	161 ± 8	182	81 ± 4	99	272
L‐(+)‐DHA_6	185	173 ± 9	182	90 ± 5	119	275
L‐(+)‐DOA_8	178	152 ± 8	175	140 ± 7	124	277
L‐(+)‐DDA_12	163	142 ± 7	165	143 ± 7	155	279
L‐(+)‐DTDA_14	162	135 ± 7	158	135 ± 7	167	276
L‐(+)‐DHDA_16	153	130 ± 7	154	120 ± 6	179	211
L‐(+)‐DODA_18	146	126 ± 6	150	124 ± 6	192	200

a) D‐ decomposition during melting.

**Figure 4:**
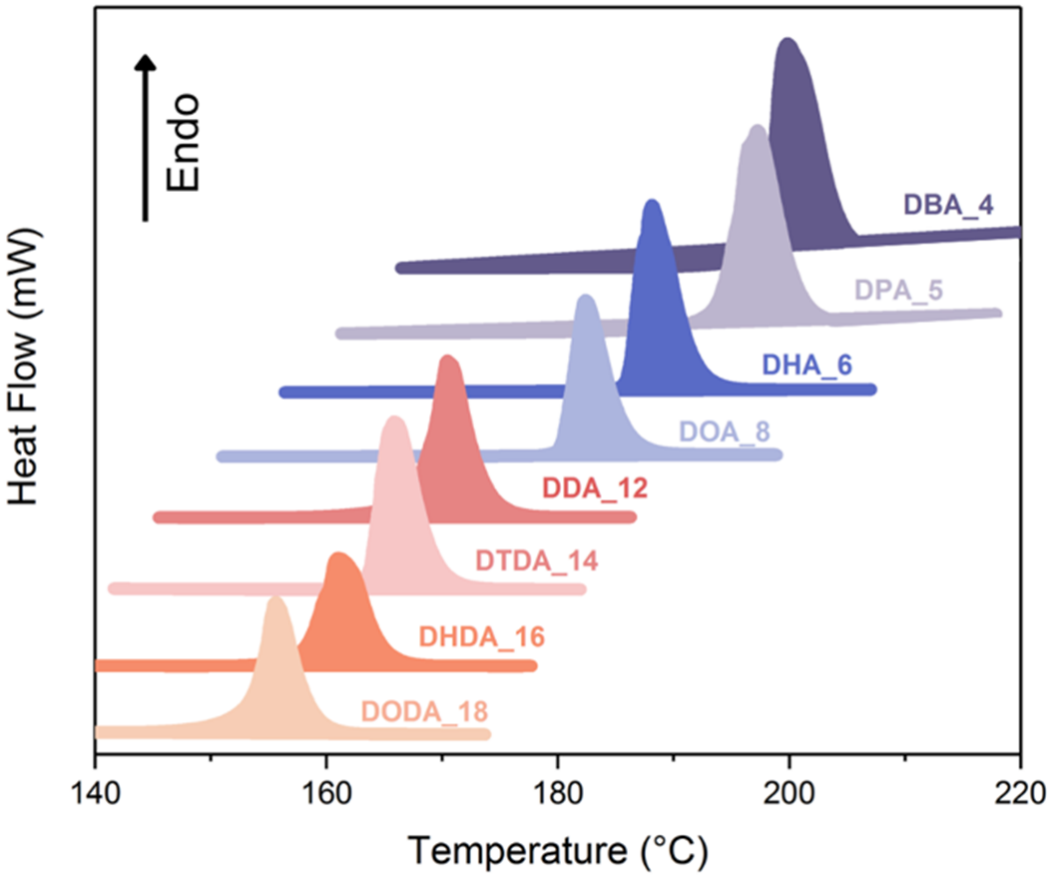
Thermal properties of L‐(+)‐TA diamide series.

Transformation of pure L‐(+)‐TA, which decomposes upon melting (*T*_m_ = 170 °C ± 2 °C, *T*_d_ = 172 °C) into amides with long alkyl chains resulted in compounds that melted between 146 and 190 °C ± 2 °C. The extension of the alkyl chain from C_4_ to C_18_ resulted in a decrease in both the melting temperature and the enthalpy of melting (**Figure** **4**). The same trend was observed previously by Poopalam et al. for fatty acid diamides.^[^ ^8^ ^]^ We observed this trend for the diesters of mucic acid, where the diester with C_12_ alkyl chains melted at 137 °C, while the ester with longer C_18_ alkyl chains melted at 124 °C.^[^ ^5^ ^]^ Contrarily, in the series of TA esters, the opposite trend was observed – with the elongation of the alkyl chain length, the *T*_m_ values increased from 67 to 82 °C for the diesters with C_12_ and C_18_ alkyl chains, respectively.^[^ ^5^ ^]^ To better understand this unexpected observation, we took a closer look into the molecular structure of these amides. Firstly, the aliphatic chain contributes to the van der Waals forces.

Secondly, the amide and hydroxyl groups provide hydrogen bonding. As the alkyl chain length increases and the van der Waals forces increase, it can be expected that both Δ*H*_f_ and *T*_m_ will increase.^[^ ^23^ ^]^ Entropy is directly tied to the heightened elasticity of the hydrocarbon chain caused by its elongation. This augmentation in degrees of freedom broadens the avenues through which thermal energy can disperse within the molecule. However, the effect of introducing multiple hydrogen‐bonding groups is more complex.^[^ ^24^ ^]^ The first hydrogen bond usually plays a dominant role in determining the crystal structure, leading to a substantial increase in melting temperature and melting enthalpy. Additional hydrogen bonds tend to have a smaller and less predictable impact on the thermal properties. This is because the geometric constraints imposed by the initial hydrogen bonds often prevent further hydrogen‐bonding groups from optimally interacting with the existing ones.^[^ ^25^, ^26^ ^]^ Moreover, hydrogen bonds may compete with dispersion forces in stabilizing the most energetically favorable crystal structure, limiting the formation of all potential interactions. Therefore, the extended H‐bonding interactions present in the series of diamides of TA and the series of esters of mucic acid most probably dictate the decrease in the melting temperature with increasing alkyl chain length, which was not observed for the esters of TA.

The investigated diamides showed significant ΔH_*f*_ values, ranging from 126 ± 6 J g^−1^ for L‐(+)‐DODA_18 to 173 ± 9 J g^−1^ for L‐(+)‐DHA_6, which were also affected by the discussed H‐bonding interactions. Interestingly, the difference between the Δ*H*_f_ of L‐(+)‐DDA_12 (142 ± 7 J g^−1^) and its analog without ‐OH groups reported in the literature (149 ± 7 J g^−1^) was only 7 J g^−1^. This phenomenon can be attributed to steric hindrance between closely located amide groups and ‐OH groups, limiting the mobility of the molecule to adopt a more favorable conformation.^[^ ^8^ ^]^ As the alkyl chain of the diamide was lengthened to 18 carbon atoms, this difference increased to 50 ± 3 J g^−1^ (Δ*H*_f_ = 126 ± 6 J g^−1^ for L‐(+)‐DODA_18 and 176 ± 9 J g^−1^ for the corresponding analog). Comparing the melting temperature of the investigated diamides with similar ones without hydroxyl groups showed that the introduction of two additional hydroxyl groups to the system increased the melting temperature by only 12 °C (for L‐(+)‐DDA_12, *T*_m_ = 171 °C ± 2 °C, while the diamide synthesized from dodecyl acid and ethyldiamine showed a *T*_m_ of 159 °C). An equally important observation is that for L‐(+)‐DPA_5, the melting point and enthalpy of melting deviated slightly from the expected trend and were quite similar to those of L‐(+)‐DBA_4. This similarity can be most probably attributed to the previously reported more efficient crystal packing of amides with an even‐number of carbon atoms in the chain.^[^ ^11^ ^]^

## 3 Understanding Thermal Transition Behavior

### 3.1 Crystallographic Study

To explain the influence of the structure of compounds on their thermal properties, crystallographic analysis is highly useful.^[^ ^5^, ^16^, ^27^ ^]^ Unfortunately, growing suitable diamide crystals proved challenging due to their long alkyl chains, which caused them to crystallize as long, thin needles. Since most hydrogen bonds originate from the diamide core, the following analysis focuses primarily on D, L, and DL‐TA crystals available in databases and described in the literature.^[^ ^28^, ^29^, ^30^ ^]^ This analysis aims to correlate the hydrogen bonds in these crystal structures with the thermal properties of diamide derivatives (meso‐TA occurs in the form of a hydrate, with no crystallographic data available for the unhydrated compound). The crystal structure of TA isomers is dominated by strong hydrogen bonds. However, the crystal packing of the isomers differs in the number of hydrogen bonds and their strength, significantly influencing the melting temperature and enthalpy of fusion.^[^ ^31^ ^]^ L‐ and D‐TA are mirror images with nearly identical bond lengths and angles. Both create hydrogen bond networks forming layers parallel to the ac plane and share a monoclinic crystal system with space group P2_1_.^[^ ^29^ ^]^ L‐(+)‐TA and D‐(−)‐TA have strong hydrogen bonds, including short bicentric bonds (≈2.6–2.9 Å, angles >159°). Their dense crystal packing, the most compact among the isomers, likely causes their higher melting points and enthalpies, as seen in L‐(+)‐DDA_12 and D‐(−)‐DDA_12.^[^ ^15^ ^]^ However, while this explains the similar melting points, it does not clarify the lower enthalpy observed for D‐(−)‐DDA_12.

The racemic mixture showed the weakest hydrogen bonds and the loosest packing, resulting in the lowest melting point being determined for DL‐DDA_12.^[^ ^30^ ^]^ These findings indicate that analyzing hydrogen bonds in the precursors can help to predict the thermal properties of their derivatives. Similarly to sugar alcohols, the arrangement of ‐OH groups in TA significantly affects the melting enthalpy and melting point,^[^ ^18^ ^]^ with the highest values being observed for L‐(+)‐TA, which shows the smallest repulsive O‐O interactions. This pattern was also observed for hexa‐esters of galactitol^[^ ^32^ ^]^ and mannitol,^[^ ^33^ ^]^ where galactitol hexastearate shows a higher Δ*H*_f_ value than mannitol hexastearate (200 and 250 J g^−1^, respectively).

### 3.2 FT‐IR Analysis

**Figure 5:**
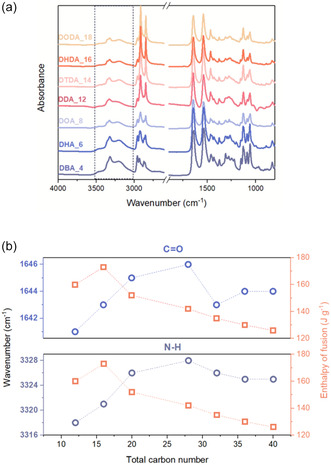
a) FT‐IR spectra of the L‐(+)‐TA diamide series, b) Shift of N—H (violet circles, ≈3322 cm^−1^), C=O (blue circles, ≈1645 cm^−1^) wavenumbers and enthalpy of fusion (squares) with increasing chain length; dashed lines are guides to aid visualization.

FT‐IR spectroscopy was conducted to further understand the relative contribution of H‐bonding and van der Waals interactions in the series of TA diamides. Changes in bond length cause shifts in the characteristic infrared absorption frequencies. To monitor the structural changes of diamides during melting, we analyzed several key absorption bands, which had been previously described in the literature: the symmetric CH_2_ stretching band (≈2920 and ≈2850 cm^−1^) to assess van der Waals interactions, and the C=O stretching (≈1650 cm^−1^), N—H bending (≈1550 cm^−1^), and N—H and O—H stretching (≈3300 cm^−1^) bands to study hydrogen bond strength.^[^ ^23^, ^34^ ^]^ Analysis revealed a shift in the N—H peak toward higher wavenumbers with increasing chain length, particularly in compounds from L‐(+)‐DBA_4 to L‐(+)‐DHA_6, indicating weakened hydrogen bonding (**Figure** **5**a). However, this shift did not correlate with the expected decrease in enthalpy. We hypothesize that the diamides partially decomposed into their constituent acid and amine components upon melting. Since the amines had much lower boiling points (ranging from 77 °C for butylamine to 178 °C for octylamine) than the melting points of their corresponding diamides, partial amine evaporation may occur during heating.

As the alkyl chain lengthened, the weight loss after three heating cycles decreased (from 80% for L‐(+)‐DBA_4 to 7% for L‐(+)‐DOA_8), supporting this hypothesis. Moreover, the observed melting enthalpy values for this group of compounds may have been artificially elevated due to the concurrent evaporation process, which is typically highly endothermic. However, this trend deviated for compounds with alkyl chains longer than 8 carbon atoms. The N‐H peak showed only minimal shifts in L‐(+)‐DOA_8 and L‐(+)‐DDA_12, reaching 3328 cm^−^^1^. In L‐(+)‐DHDA_16 and L‐(+)‐DODA_18, the peak position slightly reversed to 3325 cm^−^^1^. A similar pattern was observed for the C=O band. The N—H peak intensity decreased progressively throughout the series, further supporting the weakening of hydrogen bonds. These findings suggest that van der Waals forces become increasingly dominant as chain length increases. For larger molecules (C_14_ to C_18_), we hypothesize that the extended alkyl chains provide enhanced molecular flexibility, potentially facilitating the formation of stronger hydrogen bonds. However, this hypothesis does not account for the observed decrease in both melting temperatures and associated enthalpies for these diamides. These results suggest that the thermal properties of the compounds are governed not only by hydrogen bond strength and van der Waal forces but also by additional factors.

**Figure 6:**
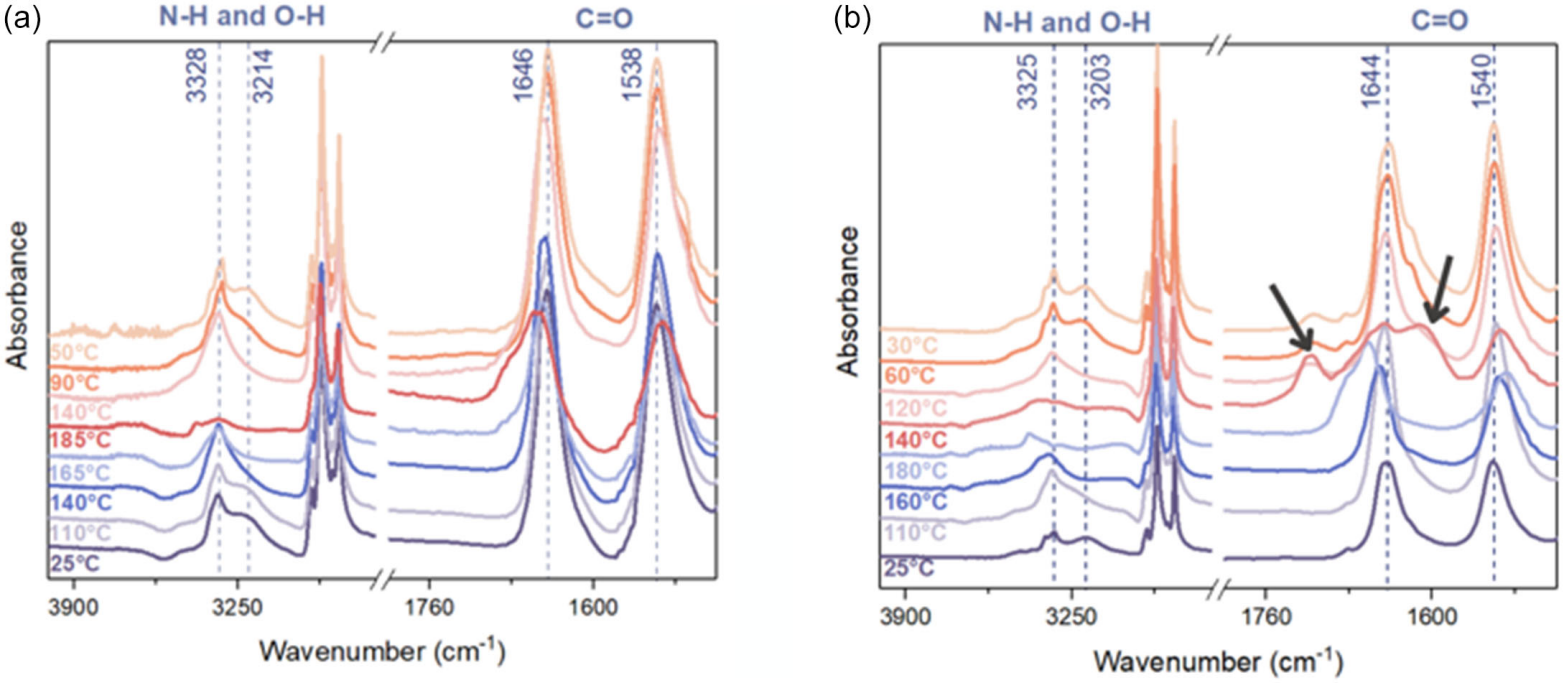
FT‐IR of a) L‐(+)‐DDA_12, b) L‐(+)‐DHDA_16.

To further investigate molecular interactions, variable temperature FT‐IR spectroscopy was employed (**Figure** **6**).

Upon heating above their respective melting temperatures, all the compounds showed a shift in the maxima of the symmetric and antisymmetric stretching vibrations of the CH_2_ band by 3–4 cm^−1^ (≈2850 cm^−1^) and 5–7 cm^−1^ (≈2920 cm^−1^) (Figure 6). These changes indicated the increased mobility of the hydrocarbon chain through hydrogen bond weakening. The C=O region is shown in Figure 6. At room temperature, signals at ≈1647 cm^−1^ were observed, originating from hydrogen‐bonded carbonyl groups. Increasing the temperature led to a shift in the C=O band to lower wavenumbers (1646 cm^−1^ for L‐(+)‐DDA_12) and peak broadening as the hydrogen bonds weakened or dissociated. Interestingly, upon cooling, these signals returned to their initial wavenumber values, demonstrating the reversibility of hydrogen bonding for L‐(+)‐DOA_8 and L‐(+)‐DDA_12. No peaks at 1740 cm^−1^, which would correspond to non‐hydrogen‐bonded C=O groups, were observed.^[^ ^35^ ^]^ For L‐(+)‐DHA_6 and L‐(+)‐DOHA_16, an additional peak at ≈1718 cm^−1^ was observed after melting (Figure S24, Supporting Information).

Definitive evidence for the presence of strong hydrogen bonds came from the analysis of the N—H and O—H stretching bands. The N—H bending absorption shifted to higher wavenumbers with increasing temperature, reflecting the weakening of the hydrogen bonds. The absence of peaks above 3580 and 3460 cm^−1^ (characteristic of free hydroxyl and free N—H groups, respectively) indicated that the hydrogen bonds remained intact throughout melting. Notably, the ratio of N—H to CH_2_ peaks decreased with increasing chain length, suggesting a greater influence of van der Waals interactions on the structure.

### 3.3 Thermal Stability

**Figure 7:**
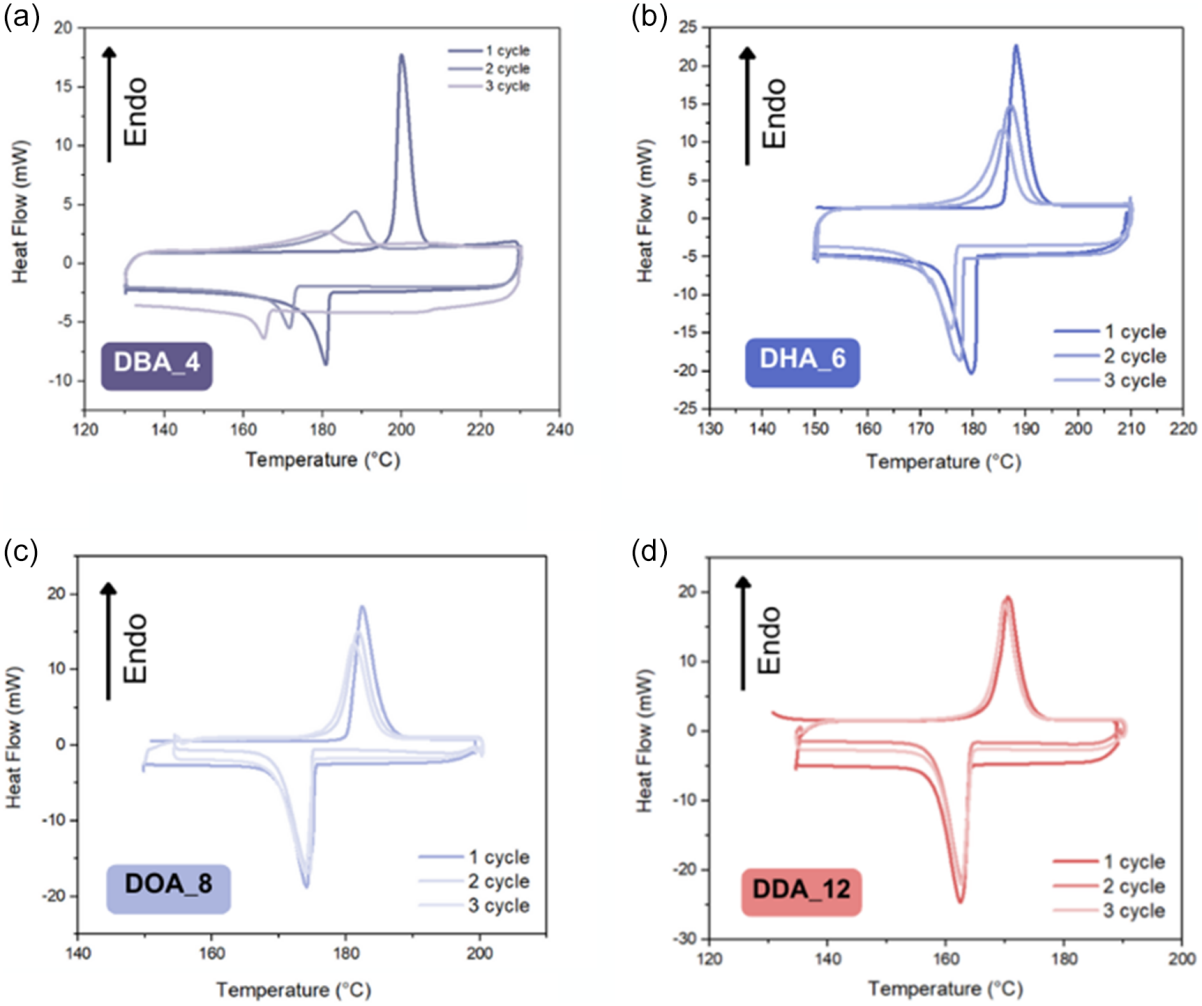
Stability a) L‐(+)‐DBA_4, b) L‐(+)‐DHA_6, c) L‐(+)‐DOA_8, and d) L‐(+)‐DDA_12 over three heating/cooling cycles.

The thermal stability of all the diamides was initially assessed through three heating and cooling cycles using DSC (Figure S26, Supporting Information). Short‐chain amides (C_4_, C_5_, C_6_, and C_8_) showed poor stability under repetitive heating and cooling conditions, as evidenced by two factors. Firstly, we observed a shift in the melting transition to lower temperatures with each heating cycle, and secondly, we noted sample mass loss after analysis compared to the initial mass. When extending the alkyl chains from C_4_ to C_12_, the stability notably improved, which was observed as reduced mass loss and smaller variations in *T*_m_ (**Figure** **7** and S26, Supporting Information).

TGA analysis was performed to investigate whether amine evaporation from partial decomposition might explain the instability of shorter‐chain amides. The boiling points of the amines with alkyl chains up to C8 are significantly lower than the melting points of their corresponding diamides (the boiling point of butylamine is 77 °C whereas the *T*_m_ of L‐(+)‐DBA_4 is 190 °C; for octylamine the boiling point is 178 °C, whereas the *T*_m_ of the L‐(+)‐DOA_8 is 178 °C). As the amides’ alkyl chain lengths increased, their melting temperature decreased. Consequently, amides derived from amines with at least C_12_ carbon atoms showed *T*_m_ values below the boiling points of the corresponding amines, which aligns with their improved stability over three heating‐cooling cycles. Interestingly, further extending the alkyl chain from C_16_ to C_18_ led to decreased stability (Figure S26, Supporting Information). For L‐(+)‐DDTA_14, no drop in enthalpy was seen after three cycles, but testing over ten cycles confirmed the diamide's instability. Chen et al. observed a similar trend with fatty amines.^[^ ^36^ ^]^ For instance, 1‐dodecylamine maintained stability over 50 heating and cooling cycles, while 1‐tetradecylamine and 1‐hexadecylamine showed a significant decrease in latent heat. The underlying mechanism of this phenomenon remains unclear. The authors suggested that during melting and freezing cycles, the PCM's crystal structure may undergo rearrangement, potentially impacting its latent heat properties.^[^ ^36^ ^]^

All the synthesized diamides exhibited high thermal stability under standard TGA experimental conditions (heating at a rate of 10 °C min^−1^), with *T*_d_ values above 200 °C (Table 1, Figure 9). The highest stability of 279 °C ± 2 °C was observed for the amide L‐(+)‐DDA_12. Unlike melting temperatures, the decomposition temperatures of the diamides showed an increasing trend with alkyl chain extension. This trend can be attributed to the increasing contribution of van der Waals interactions between the alkyl chains and the higher molecular weight of the compounds. However, the compounds with the longest alkyl chains (L‐(+)‐DHDA_16 and L‐(+)‐DODA_18) deviated from this trend, showing decreased decomposition temperatures. Similar thermal behavior was previously observed for mucic acid diesters.^[^ ^5^ ^]^

**Figure 8:**
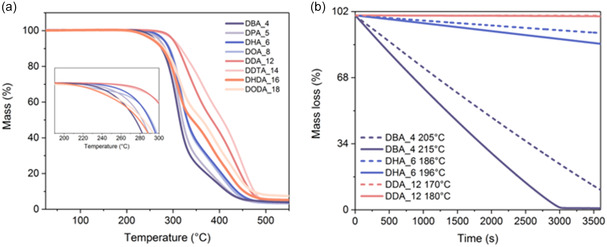
a) TGA and b) TGA isothermal of L‐(+)‐TA diamide.

Interestingly, the TGA analysis did not confirm the mass loss at the melting point for the diamides with the shortest alkyl chains up to C_8_ that was observed during DSC analysis. To better understand this phenomenon, we performed isothermal TGA analysis for the diamides with alkyl chain lengths of C_4_, C_6_, and C_12_, at their melting point, and 10 °C above the melting point, for 1 h (**Figure** **8**b). The short‐chain amides L‐(+)‐DBA_4 and L‐(+)‐DHA_6 exhibited noticeable mass loss at their melting temperature and at 10 °C above it, confirming the potential decomposition of the amide and the resulting amine evaporation during the analysis, while L‐(+)‐DDA_12 showed no decomposition in the isothermal settings. The MS spectrum registered for the L‐(+)‐DBA_4 sample after DSC analysis remained unchanged, which also suggested that only the undegraded amide residue remained after evaporation of the amine and acid, in line with the observed significant sample mass loss.

**Figure 9:**
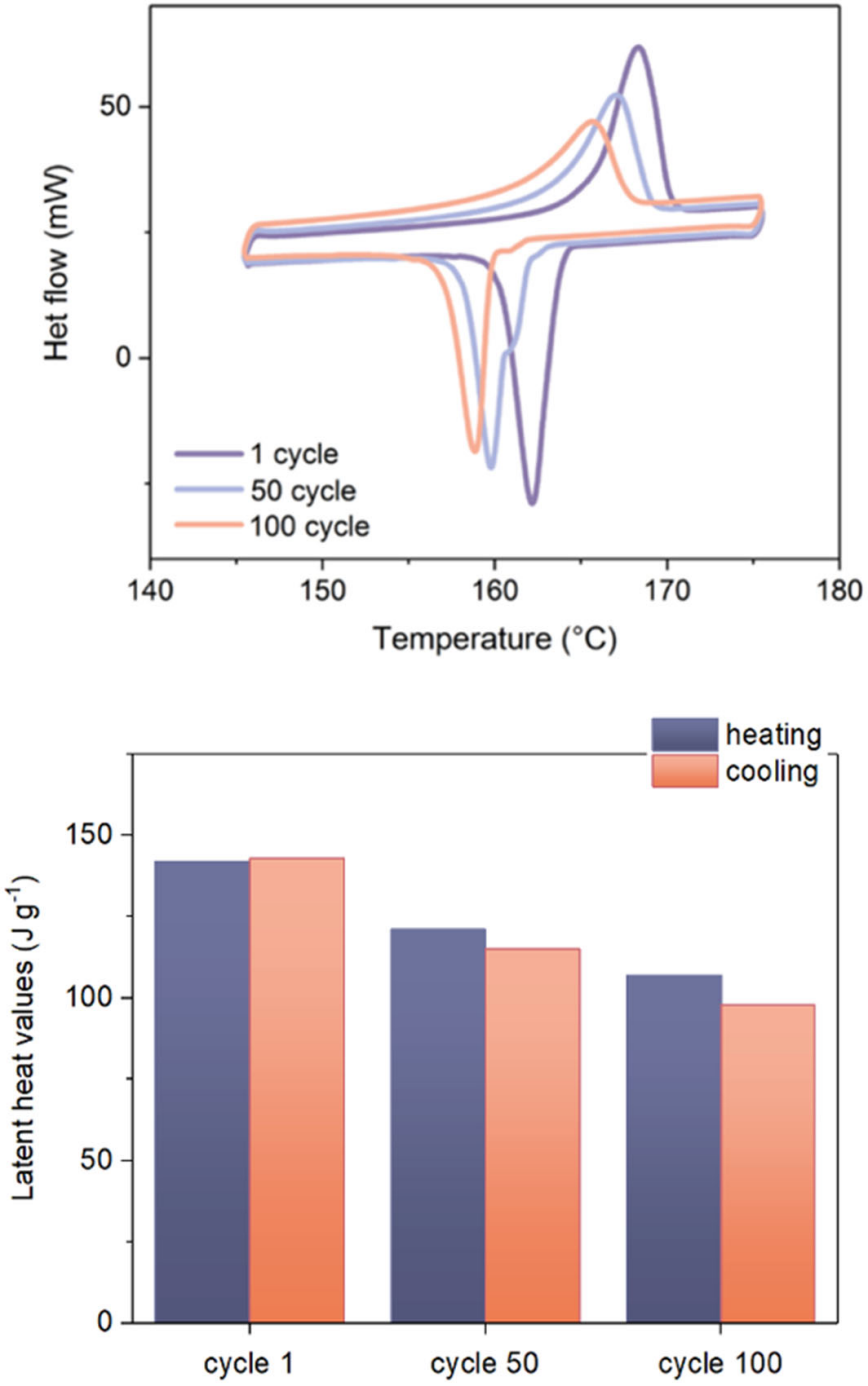
Cycling stability of L‐(+)‐DDA_12. DSC traces showing 1st, 50th and 100th cycle and associated enthalpies of fusion.

Given the initially observed improved stability of amides with increasing alkyl chain length (Figure 7), L‐(+)‐DDA_12, which exhibited the highest Δ*H*_*f*_ value, was selected for long‐term stability testing (repetitive heating and cooling of an 8 mg sample over 100 cycles over a temperature range of 145–175 °C ± 2 °C). Despite the high stability observed during three heating‐cooling cycles, the diamide displayed a notable Δ*H*_f_ reduction of 25 % over 100 cycles, decreasing from 142 ± 7 to 107 ± 5 J g^−1^ (**Figure** **9**). The decomposition at elevated temperature was most likely promoted by trace amounts of water, polyhydroxy compounds being prone to hydrolysis to starting materials^[^ ^16^, ^31^ ^]^

## 4 Conclusion

In this study, we investigated the thermal properties of TA diamides synthesized via a straightforward procedure from an inexpensive, available and bioderived precursor. The TA‐derived amides exhibited higher *T*_m_ values (between 146 and 190 °C (±2 °C)) than their corresponding ester derivatives (67 and 97 °C). The compounds showed high enthalpies of fusion, ranging from 126 ± 6 to 173 ± 9 J g^−1^. Among TA isomers, the L‐(+)‐DDA_12 isomer showed the highest stability, as confirmed by DSC and TGA analysis. Similarly to TA‐derived esters, the amides demonstrated minimal supercooling, with a temperature difference of 6–15 °C between melting and crystallization.

TA, with its hydroxyl group‐rich structure, provides additional H‐bonding interactions to the derived PCMs, translating to high *T*_m_ and high Δ*H*_f_ as recently demonstrated for derived esters (significant improvement was achieved in relation to similar compounds lacking OH groups). Further increase in the contribution of the H‐bonds through the incorporation of amide groups into the structure resulted in higher melting temperatures. Nevertheless, the analysis of the TA‐derived amides with *T*_m_ values over 153 °C revealed their limited stability over repetitive melting and crystallization (25 % decrease in Δ*H*_f_ over 100 cycles). Even though in this study we showed that tartaric‐derived amides do not have the desired stability that is expected for PCMs, this investigation is an important step toward the development of new biobased PCMs. Future studies should focus on structural modifications to enhance the thermal cycling stability, while maintaining the advantages of using renewable resources, as the development of sustainable PCMs from biobased feedstocks remains crucial for addressing the growing demand for green energy storage solutions.

## Supporting Information

The authors have cited additional references within the Supporting Information.^[37]^

## Conflict of Interest

The authors declare no conflict of interest.

## Supporting information

Supplementary Material
