# Supplementary material for: Perspectives and Limitations of Tartaric Acid Diamides as Phase Change Materials for Sustainable Heat Applications
Source: ChemSusChem. 2025 Apr 16;18(13):e202500145. doi: 10.1002/cssc.202500145 (PMC12232102; doi:10.1002/cssc.202500145)
Supplement: Supplementary file 1 — Supplementary Material [file CSSC-18-e202500145-s001.pdf]

Supplementary materials for

Perspectives and limitations of tartaric acid diamides as phase change materials for sustainable heat use

Magdalena Gwóźdź,[a] Natalia Siodłak,[a] Anna Chrobok,[a] Karolina Matuszek,[*b] and Alina Brzeczek-Szafran[*a]

[a] Silesian University of Technology, Faculty of Chemistry, Department of Organic Chemical Technology and Petrochemistry, Gliwice, Poland
[b] Monash University, School of Chemistry, Melbourne, Australia

Correspondence to: Karolina.Matuszek@monash.edu, Alina.Brzeczek-Szafran@polsl.pl

List of contents

LIST OF SCHEME AND FIGURES **2**

LIST OF TABLES **3**

MATERIALS AND METHODS **3**

MATERIALS 3
GENERAL PROCEDURE OF SYNTHESIS TARTARIC ACID DIMETHYL ESTER 3
GENERAL PROCEDURE OF SYNTHESIS TARTARIC ACID AMIDES 4

CHARACTERISATION **7**

NUCLEAR MAGNETIC RESONANCE 7
MASS SPECTROMETRY 7
THERMOGRAVIMETRIC ANALYSIS (TGA) 8
DIFFERENTIAL SCANNING CALORIMETRY (DSC) 8
FOURIER-TRANSFORM INFRARED SPECTROSCOPY (FT-IR) 8

NMR SPECTRA **9**

FT-IR ANALYSIS **20**

TGA **21**

DSC **22**

LITERATURE **22**

List of scheme and figures

Scheme S1. Synthesis of tartaric acid diamide 3

Figure S1 ^{1}H NMR spectrum of L-(+)-tartaric acid dimethyl ester 9
Figure S 2 ^{1}H NMR spectrum of D-(-)-Tartaric acid dimethyl ester 9
Figure S 3 ^{1}H NMR spectrum of meso-tartaric acid dimethyl ester 10
Figure S 4 1H NMR spectrum of DL-tartaric acid dimethyl ester 10
Figure S 5 ^{1}H NMR spectrum of L-(+)-tartaric acid didodecyl amide 11
Figure S 6 ^{13}C NMR spectrum of L-(+)-tartaric acid Didodecyl amide 11
Figure S 7 ^{1}H NMR spectrum of D-(-)-tartaric acid didodecyl amide 12
Figure S 8 ^{13}C NMR spectrum of D-(-)-tartaric acid didodecyl amide 12
Figure S 9 ^{1}H NMR spectrum of DL-tartaric acid didodecyl amide 13
Figure S 10 ^{13}C NMR spectrum of DL-tartaric acid didodecyl amide 13
Figure S 11 ^{1}H NMR spectrum of meso-tartaric acid dodecyl amide 14
Figure S 12 ^{13}C NMR spectrum of meso-tartaric acid dodecyl amide 14
Figure S13 ^{1}H NMR spectrum of L-(+)-tartaric acid dibutyl amide 15
Figure S 14 ^{13}C NMR spectrum of L-(+)-tartaric acid dibutyl amide 15
Figure S 15 ^{1}H NMR spectrum of L-(+)-tartaric acid dipentyl amide 16
Figure S 16 ^{13}C NMR spectrum of L-(+)-tartaric acid dipentyl amide 16
Figure S 17 ^{1}H NMR spectrum of L-(+)-tartaric acid dihexyl amide 17
Figure S 18 ^{13}C NMR spectrum of L-(+)-tartaric acid Dihexyl amide 17
Figure S 19 ^{1}H NMR spectrum of L-(+)-tartaric acid dioctyl amide 18
Figure S 20 ^{13}C NMR spectrum of L-(+)-tartaric acid dioctyl amide 18
Figure S 21 ^{1}H NMR spectrum of L-(+)-tartaric acid ditetradecyl amide 19
Figure S 22 1H NMR spectrum of L-(+)-tartaric acid dihexadecyl amide 19
Figure S 23 ^{1}H NMR spectrum of L-(+)-tartaric acid dioctadecyl amide 20
Figure S 24 FT-IR of L-(+)-DHA_6 and L-(+)-DDHA_16 20
Figure S 25 TGA and DTGA of L-(+)-tartaric acid diamides 21
Figure S 26 DSC of L-(+)-tartaric acid diamides 22

List of tables

Materials and methods

Materials

The sugar acid diamide of L-(+)-Tartaric acid (L-(+)-TA, ≥99.5%), D-(-)-Tartaric acid (D-(-)-TA, >99.5%), DL-Tartaric acid (DL-TA, >99.5%), meso-Tartaric acid monohydrate (meso-TA, ≥97%), were synthesized using 1-butylamine (>98%), 1-penthylamine (>99%), 1-hexylamine (>99%), 1-octylamine (for synthesis), 1-dodecylamine (>98%), 1-tetradecylamine (>95%), 1-hexadecylamine (for synthesis) and 1-octadecylamine (for synthesis), from Sigma-Aldrich. For the esterification reaction *p*-toluenesulfonic acid (p-TSA) and methanol commercially available from Merck were used. For crystallization ethanol and chloroform were used. All the reagents were used without purification.

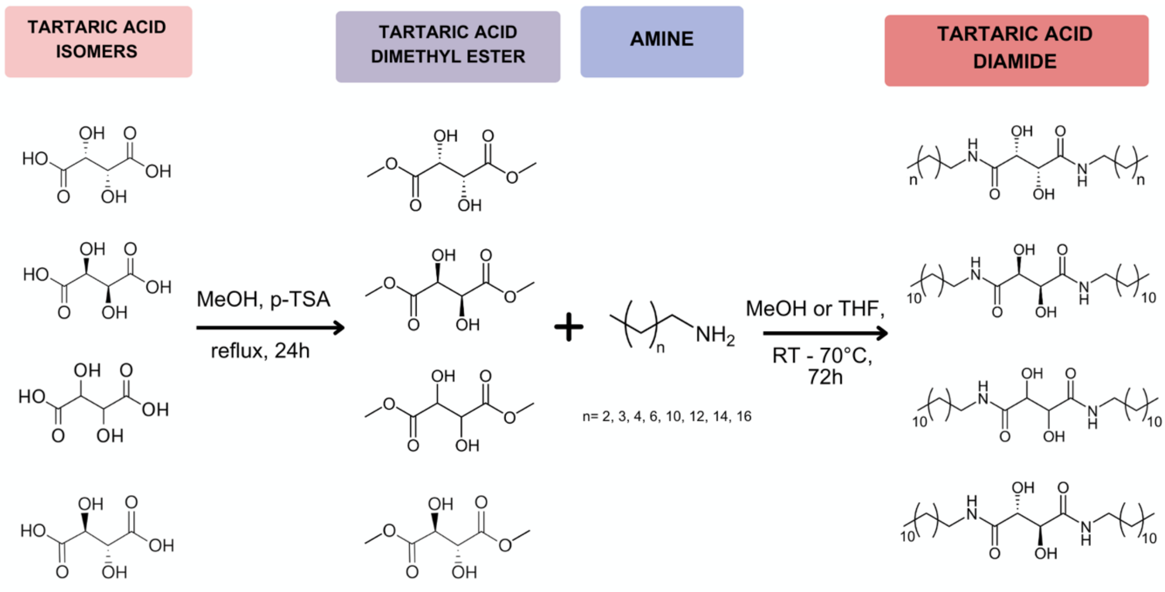

Scheme S1. Synthesis of tartaric acid diamides

General procedure of synthesis tartaric acid dimethyl ester

TA isomer (2.05 g, 14 mmol), *p*-TSA (0.13 g, 0.6 mmol) and methanol (24 mL) were refluxed for 24 h. Upon completion, the reaction was neutralized by the addition of sodium bicarbonate (0.150 g) and the resulting salt was separated by filtration. The solvent was evaporated affording tartaric acid dimethyl ester. The product was used in subsequent steps without further purification.

General procedure of synthesis tartaric acid amides

The previously synthesized tartaric acid dimethyl ester (1g, 5,61 mmol) and solvent (methanol or THF) (2.5 mL were introduced into a 25 cm^3 round-bottom flask equipped with a reflux condenser, followed by the addition of the amine (14,03 mmol). The reaction was stirred at room temperature for 72 h (for butylamine and pentylamine at 60°C). Upon completion, the product was isolated by vacuum filtration using a Büchner funnel, followed by recrystallization from ethanol and next chloroform. After vacuum-drying on a Schlenk line for 24 h, the products were obtained as white, loose crystalline precipitates.

L-(+)-Tartaric acid dimethyl ester

$C_6H_{10}O_6$; 178.14 g/mol; yield: 88.9%, transparent thick liquid

^{1}H NMR (400 MHz, DMSO) δ 5.26 (s, 1H), 4.41 (s, 1H), 3.66 (s, 3H)

ESI_MS: m/z $[M+H]^+$ calcd.: 178.0477, found: 178.0482, $[M+Na]^+$calcd.: 201.0375, found: 201.0379

D-(-)-Tartaric acid dimethyl ester

$C_6H_{10}O_6$; 178.14 g/mol; yield: 85.3%, transparent crystal

^{1}H NMR (400 MHz, DMSO) δ 5.66 – 5.44 (m, 1H), 4.41 (d, J = 5.6 Hz, 1H), 3.66 (s, 3H

ESI_MS: m/z $[M+H]^+$ calcd.: 178.0477, found: 178.0491, $[M+Na]^+$calcd.: 201.0375, found: 201.0369

DL- Tartaric acid dimethyl ester

$C_6H_{10}O_6$; 178.14 g/mol; yield: 81.6%, transparent crystal

^{1}H NMR (400 MHz, CD_3OD) δ 4.56 (s, 1H), 3.78 (s, 3H)

ESI_MS: m/z $[M+H]^+$ calcd.: 178.0477, found: 178.0480, $[M+Na]^+$calcd.: 201.0375, found: 201.0379

meso-Tartaric acid dimethyl ester

$C_6H_{10}O_6$; 178.14 g/mol; yield: 75.9%, white solid

^{1}H NMR (400 MHz, DMSO) δ 5.80 (td, J = 4.2, 1.7 Hz, 1H), 4.42 – 4.13 (m, 1H), 3.62 (s, 3H)

ESI_MS: m/z $[M+H]^+$ calcd.: 178.0477, found: 178.0493, $[M+Na]^+$calcd.: 201.0375, found: 201.0386

L-(+)-Tartaric acid didodecyl amide

$C_{28}H_{56}N_2O_4$; 484.75 g/mol; yield: 52.4%; white crystalline solid; T_t =171 °C

^{1}H NMR (400 MHz, $CDCl_3$) δ 7.08 (s, 1H), 4.23 (s, 1H), 3.24 (d, *J* = 7.4 Hz, 2H), 1.52 – 1.43 (m, 2H), 1.26 (s, 24H), 0.88 (t, *J* = 6.9 Hz, 3H).

^{13}C NMR (101 MHz, $CDCl_3$) δ 173.91, 69.95, 39.09, 31.92, 29.66, 29.64, 29.60, 29.53, 29.36, 29.25, 26.77, 22.69, 14.12

ESI_MS: m/z $[M+H]^+$ calcd.: 485.4312, found: 485.4321 $[M+Na]^+$calcd.: 507.404, found: 507.4042

D-(-)-Tartaric acid didodecyl amide

$C_{28}H_{56}N_2O_4$; 484.75 g/mol; yield: 50.1%; white crystalline solid; T_t =166 °C

^{1}H NMR (400 MHz, $CDCl_3$) δ 6.35 (t, J = 6.0 Hz, 1H), 4.76 (d, J = 7.2 Hz, 1H), 3.54 – 3.48 (m, 2H), 2.52 (dt, J = 8.7, 6.4 Hz, 2H), 0.77 (t, J = 7.0 Hz, 1H), 0.56 (d, J = 5.2 Hz, 7H), 0.53 (s, 10H), 0.20 – 0.12 (m, 2H).

^{13}C NMR (101 MHz, $CDCl_3$) δ 173.88, 77.18, 69.91, 39.05, 31.88, 29.62, 29.59, 29.56, 29.49, 29.32, 29.21, 26.73, 22.65, 14.07.

ESI_MS: m/z $[M+H]^+$ calcd.: 485.4312, found: 485.4323 $[M+Na]^+$calcd.: 507.404, found: 507.4047

DL-Tartaric acid didodecyl amide

$C_{28}H_{56}N_2O_4$; 484.75 g/mol; yield: 54.6%; white crystalline solid; T_t =153 °C

^{1}H NMR (400 MHz, $CDCl_3$) δ 7.01 (t, J = 5.7 Hz, 1H), 5.46 – 5.37 (m, 1H), 4.21 – 4.12 (m, 1H), 3.24 – 3.11 (m, 2H), 1.43 (s, 2H), 1.40 (dd, J = 21.3, 14.5 Hz, 1H), 1.27 – 1.18 (m, 9H), 1.19 (s, 11H), 0.85 – 0.77 (m, 3H).

^{13}C NMR (101 MHz, $CDCl_3$) δ 173.89, 77.18, 69.91, 39.05, 31.88, 29.62, 29.60, 29.56, 29.49, 29.32, 29.21, 26.73, 22.65, 14.08.

ESI_MS: m/z $[M+H]^+$ calcd.: 485.4312, found: 485.4321 $[M+Na]^+$calcd.: 507.404, found: 507.4050

meso-Tartaric acid didodecyl amide

$C_{28}H_{56}N_2O_4$; 484.75 g/mol; yield: 45.4%; white solid; T_t =138°C

^{1}H NMR (400 MHz, $CDCl_3$) δ 7.05 (s, 1H), 5.67 (s, 1H), 3.98 (s, 1H), 3.27 (qd, J = 7.1, 2.3 Hz, 2H), 1.37 – 1.18 (m, 19H), 0.94 – 0.80 (m, 3H).

^{13}C NMR (101 MHz, $CDCl_3$) δ 172.87, 70.12, 39.11, 31.87, 29.59, 29.58, 29.53, 29.47, 29.31, 29.28, 29.19, 26.80, 22.65, 14.08.

ESI_MS: m/z $[M+H]^+$ calcd.: 485.4312, found: 485.4321 $[M+Na]^+$calcd.: 507.404, found: 507.4056

L-(+)-Tartaric acid dibutyl amide

$C_{12}H_{24}N_2O_4$; 260.18 g/mol; yield: 19.8% white crystalline solid; T_t = 201 °C (lit. 196 °C)[1]

^{1}H NMR (400 MHz, $CDCl_3$) δ 7.09 (s, 1H), 5.47 (s, 1H), 4.25 (s, 1H), 3.26 (d, *J* = 7.0 Hz, 2H), 1.50 (dd, *J* = 8.1, 7.0 Hz, 2H), 1.34 (dd, *J* = 15.3, 7.3 Hz, 2H), 0.92 (t, *J* = 7.3 Hz, 3H).

^{13}C NMR (101 MHz, $cdcl_3$) δ 169.09, 65.22, 33.95, 26.58, 15.09, 8.85.

ESI_MS: m/z $[M+H]^+$ calcd.: 261,1814, found: 261,1831

L-(+)-Tartaric acid dipentyl amide

$C_{14}H_{28}N_2O_4$; 288.38 g/mol; yield: 40.8%, white crystalline solid; T_t = 198 °C

^{1}H NMR (400 MHz, $CDCl_3$) δ 7.09 (s, 1H), 4.24 (s, 1H), 3.25 (d, *J* = 7.2 Hz, 2H), 1.50 (d, *J* = 7.1 Hz, 2H), 1.38 – 1.22 (m, 5H), 0.90 (t, *J* = 7.0 Hz, 3H).

^{13}C NMR (101 MHz, $CDCl_3$) δ 173.86, 70.03, 39.05, 29.02, 28.86, 22.27, 13.93.

ESI_MS: m/z $[M+H]^+$ calcd.: 289.2121, found: 289.2112, $[M+Na]^+$calcd.: 311.1849, found: 311.1929

L-(+)-Tartaric acid dihexyl amide

$C_{16}H_{32}N_2O_4$; 316.44 g/mol; yield: 38.0%; white crystalline solid; T_t = 186 °C (lit. 184 °C)[1]

^{1}H NMR (400 MHz, $CDCl_3$) δ 7.08 (s, 1H), 5.47 (s, 1H), 4.24 (s, 1H), 3.40 – 3.05 (m, 2H), 1.55 – 1.38 (m, 2H), 1.41 – 1.18 (m, 6H), 0.88 (t, *J* = 6.8 Hz, 3H).

^{13}C NMR (101 MHz, $cdcl_3$) δ 173.89, 69.98, 39.09, 31.40, 29.31, 26.42, 22.52, 14.00.

ESI_MS: m/z $[M+H]^+$ calcd.: 317.2434, found: 317.2281, $[M+Na]^+$calcd.: 339.2162, found: 339.2083

L-(+)-Tartaric acid dioctyl amide

$C_{20}H_{40}N_2O_4$; 372.54 g/mol; yield: 47.2%; white crystalline solid; T_t = 182 °C (lit. 178 °C)[1]

^{1}H NMR (400 MHz, $CDCl_3$) δ 7.08 (s, 1H), 5.47 (d, *J* = 7.2 Hz, 1H), 4.24 (d, *J* = 6.5 Hz, 1H), 3.24 (dd, *J* = 13.8, 6.6 Hz, 2H), 1.54 – 1.44 (m, 2H), 1.28 (s, 8H), 0.88 (t, *J* = 6.7 Hz, 3H).

^{13}C NMR (101 MHz, $CDCl_3$) δ 173.89, 69.98, 39.10, 31.79, 29.36, 29.19, 29.18, 26.77, 22.64, 14.08

ESI_MS: m/z $[M+Na]^+$calcd.: 395.2780, found: 395.2546

L-(+)-Tartaric acid ditetradecyl amine

$C_{32}H_{64}N_2O_4$; 540.86 g/mol; yield: 61.7%; white crystalline solid; T_t =165 °C

^{1}H NMR (400 MHz, $CDCl_3$) δ 7.07 (s, 1H), 4.17 (s, 1H), 3.20 – 3.13 (q, 2H), 1.45 – 1.41 (m, 2H), 1.19 (m, 23H), 0.79 – 0.83 (t, 3H).

ESI_MS: m/z $[M+H]^+$ calcd.: 541.4944, found: 541.4936, $[M+Na]^+$calcd.: 563.4764, found: 563.4752

L-(+)-Tartaric acid dihexadecyl amine

$C_{36}H_{72}N_2O_4$; 596.97 g/mol; yield: 63.4%; white crystalline solid; T_t =161 °C

^{1}H NMR (400 MHz, $CDCl_3$) δ 7.01 (s, 1H), 5.43 (d, *J* = 7.4 Hz, 1H), 4.16 (d, *J* = 6.5 Hz, 1H), 3.17 (q, *J* = 6.7 Hz, 2H), 1.42 (d, *J* = 7.3 Hz, 4H), 1.19 (s, 56H), 0.80 (q, *J* = 8.4 Hz, 11H).

ESI_MS: m/z $[M+H]^+$ calcd.: 597.5570, found: 597.5555, $[M+Na]^+$calcd.: 619.5390, found: 619.5375

L-(+)-Tartaric acid dioctadecyl amine

$C_{40}H_{80}N_2O_4$; 653.07 g/mol; yield: 70.5%; white crystalline solid; T_t =156 °C

^{1}H NMR (400 MHz, $CDCl_3$) δ 5.49 (d, J = 8.2 Hz, 1H), 4.23 (d, J = 7.9 Hz, 1H), 3.24 (q, J = 6.8 Hz, 2H), 1.26 (s, 35H), 0.97 – 0.86 (m, 3H).

ESI_MS: m/z $[M+H]^+$ calcd.: 653.6196, found: 653.6177, $[M+Na]^+$ calcd.: 675.6016, found: 675.5998

Characterisation

Nuclear magnetic resonance

The structures were confirmed by ^{1}H NMR and ^{13}C NMR spectroscopy, using a 400 MHz Agilent spectrometer or Bruker Avance III NMR Spectrometer. Chemical shifts (ppm) were reported relative to the internal standard tetramethylsilane (TMS). Chemical shifts (ppm) were referenced to the internal standard tetramethylsilane (TMS). The solvents employed for the measurements included $CDCl_3$, CD_3OD and DMSO- d_6.

Mass spectrometry

High-resolution mass spectrometry was performed using a Waters Xevo G2 QTof mass spectrometer equipped with an ESI source operating in positive-ion mode. The molecular mass and composition were accurately determined with the assistance of MassLynx software.

Thermogravimetric analysis (TGA)

Thermogravimetric analysis was carried out with a Mettler Toledo TGA/DSC 1 STARe system. Sample masses ranging from 5 mg to 20 mg were placed in hermetically sealed aluminum pans. The temperature was raised from 25 °C to 550 °C at a constant heating rate of 10 °C/min, while a nitrogen flow rate of 30 mL/min was sustained throughout the analysis.

Differential scanning calorimetry (DSC)

Phase transition temperatures, including melting (T_m), crystallization (T_c) and heat of fusion (ΔH_f), were analyzed using a TA Q200 differential scanning calorimeter (TA Instruments). The instrument was calibrated using indium (T_m = 156 °C, ΔH_f = 28.45 J g^{-1}) and cyclohexane (T_m = 8 °C) standards. Measurements were conducted in triplicate under a nitrogen atmosphere with sample weights of 2–10 mg and a heating rate of 10 °C/min. For stable compounds, data from the second heating and cooling cycles were considered, whereas for compounds that decomposed, the first cycle data were utilized. Phase transition temperatures (T_m and T_c) were identified from the onset temperature, and ΔH_f was calculated by integrating the area under the melting peak using Pyris software. The uncertainties of the DSC measurements were determined through triplicate runs. Analysis of these repeated measurements indicated that the error associated with melting transition temperatures was ±2°C, while the uncertainty for enthalpy of fusion values was within 5% of the reported values.

Fourier-transform infrared spectroscopy (FT-IR)

Transmission FT-IR spectra of the tartaric acid diamide samples were recorded at room temperature (RT) and after melting using a Mettler Toledo iC10 Fourier Transform IR spectrometer. The spectrometer was equipped with a liquid-nitrogen-cooled mercury-cadmium-telluride (MCT) detector. The samples were applied to a diamond ATR crystal for measurement. Data were collected across the spectral range of 4000–650 cm^{-1}, with a resolution of 2 cm^{-1}, and averaged over 64 scans.

NMR spectra

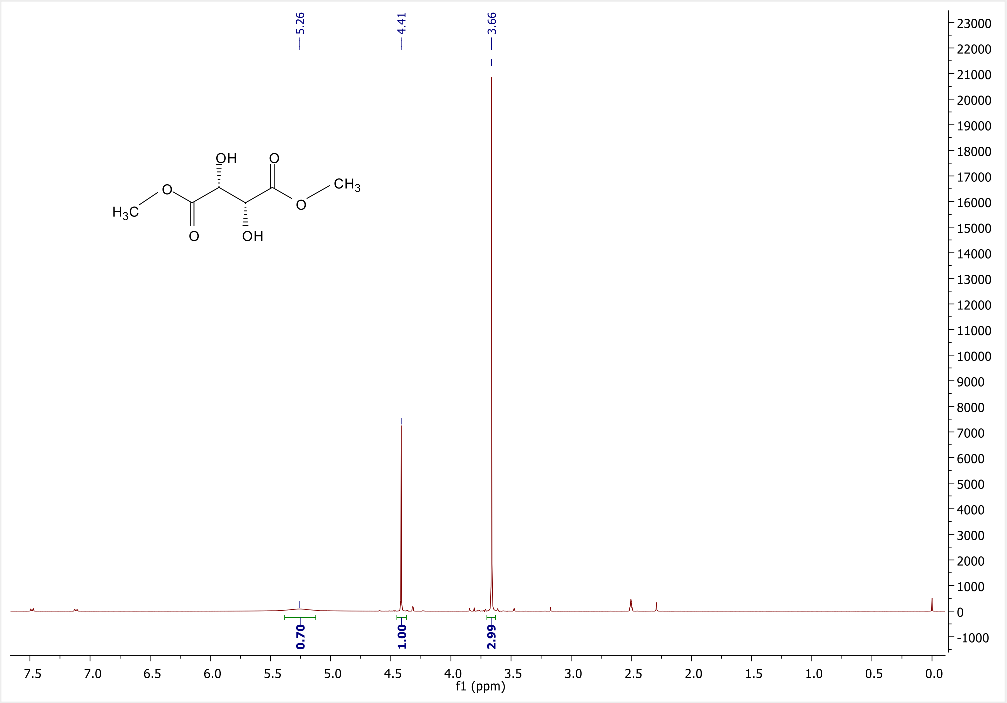

Figure S1 ^{1}H NMR spectrum of L-(+)-tartaric acid dimethyl ester

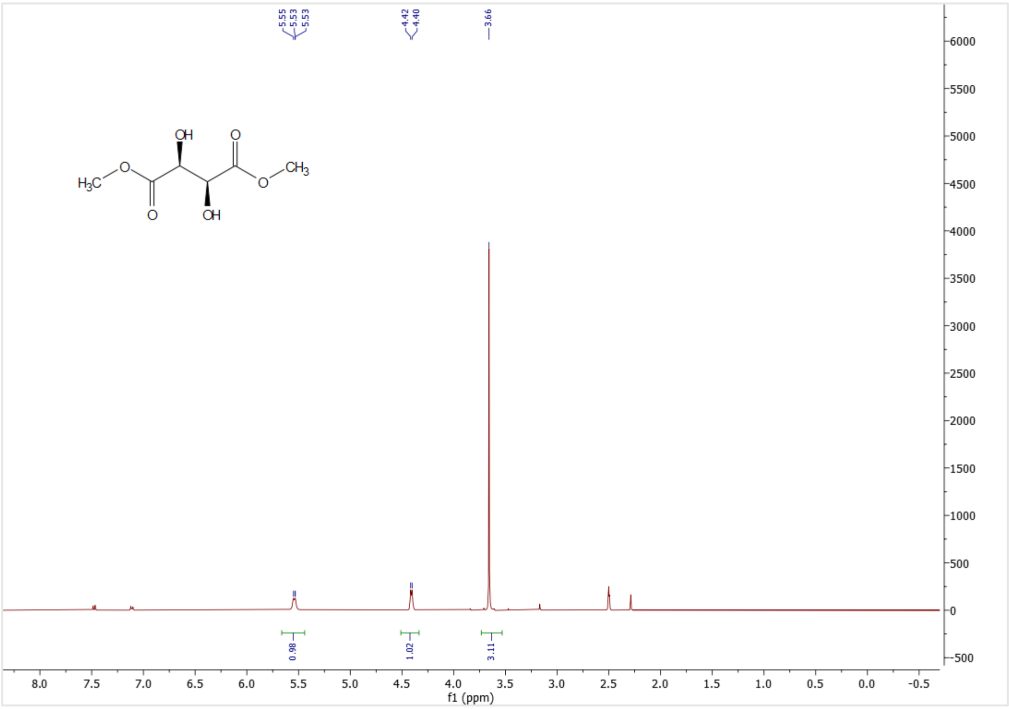

Figure S 2 ^{1}H NMR spectrum of D-(-)-Tartaric acid dimethyl ester

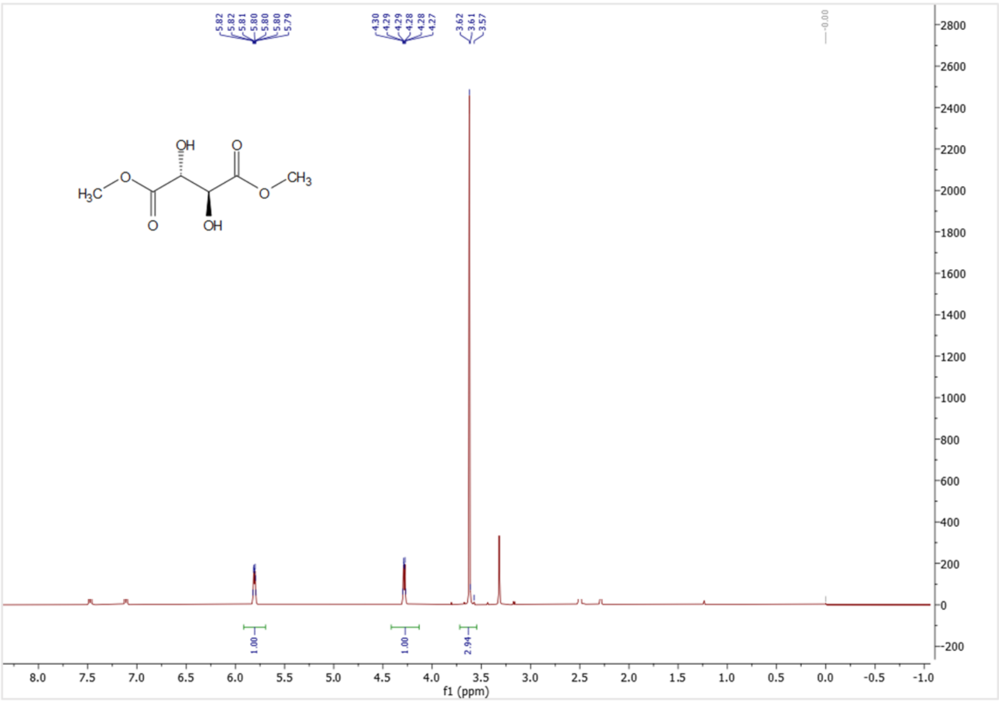

Figure S 3 ^{1}H NMR spectrum of meso-tartaric acid dimethyl ester

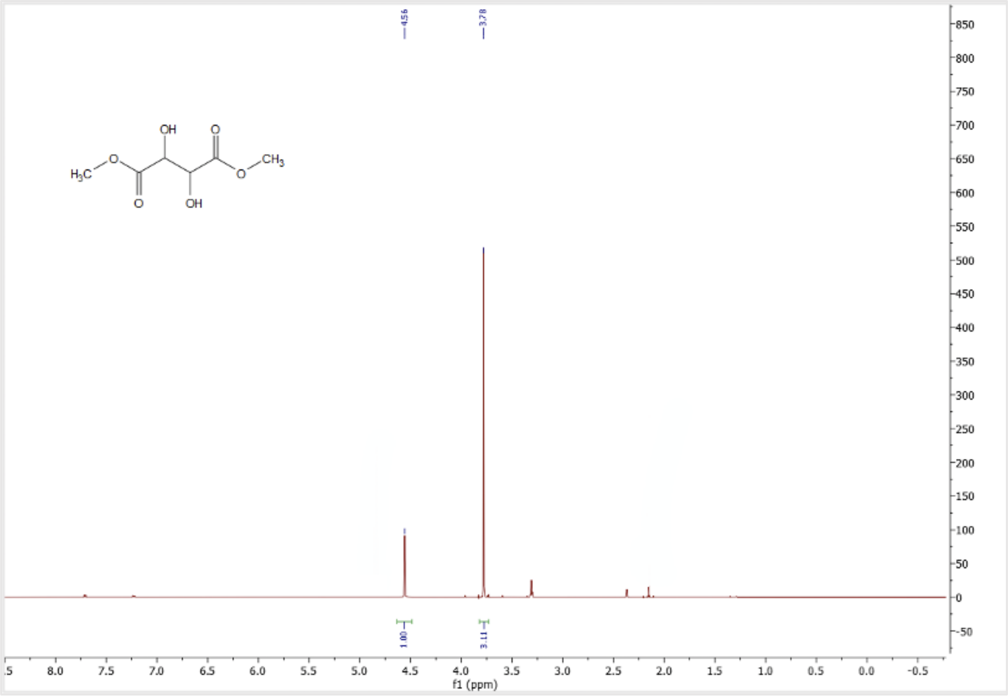

Figure S 4 1H NMR spectrum of DL-tartaric acid dimethyl ester

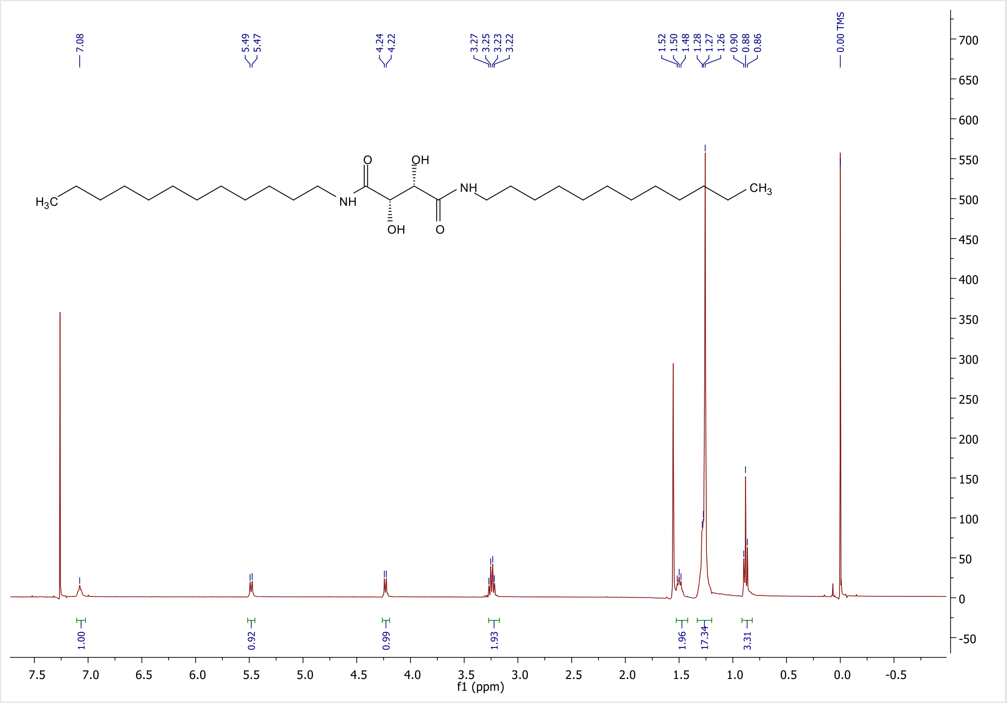

Figure S 5 ^{1}H NMR spectrum of L-(+)-tartaric acid didodecyl amide

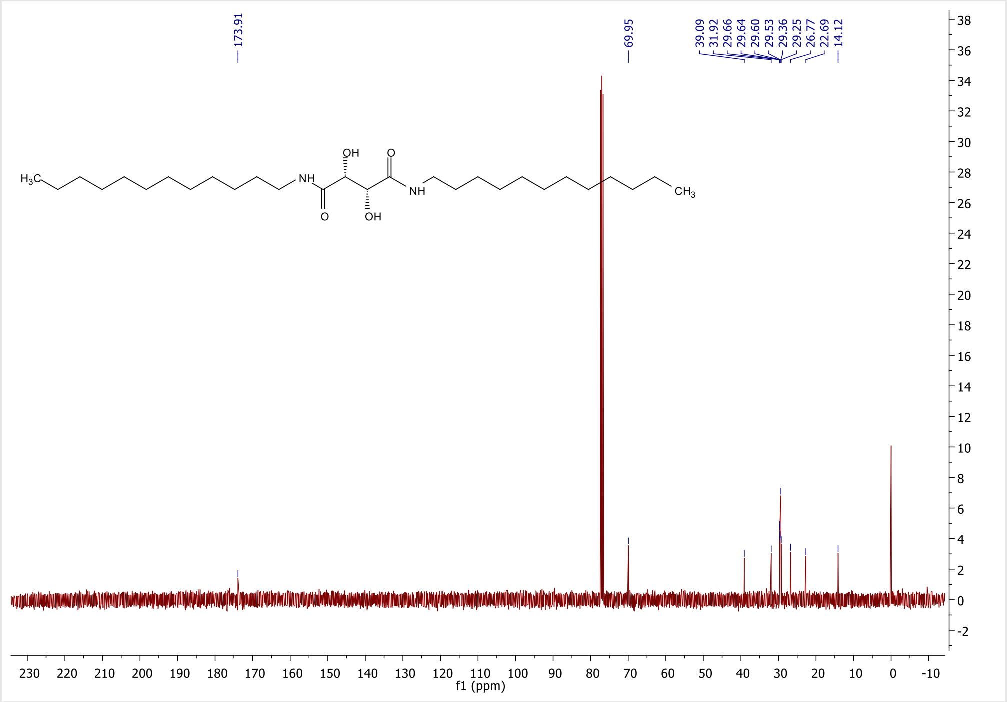

Figure S 1 ^{13}C NMR spectrum of L-(+)-tartaric acid Didodecyl amide

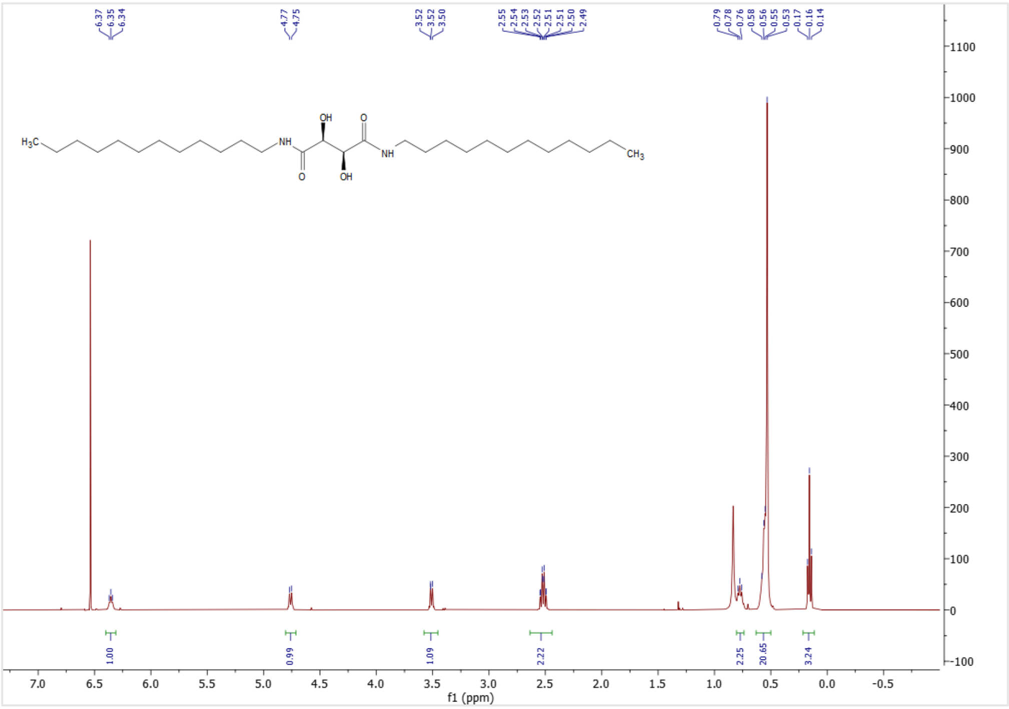

Figure S 7 ^{1}H NMR spectrum of D-(-)-tartaric acid didodecyl amide

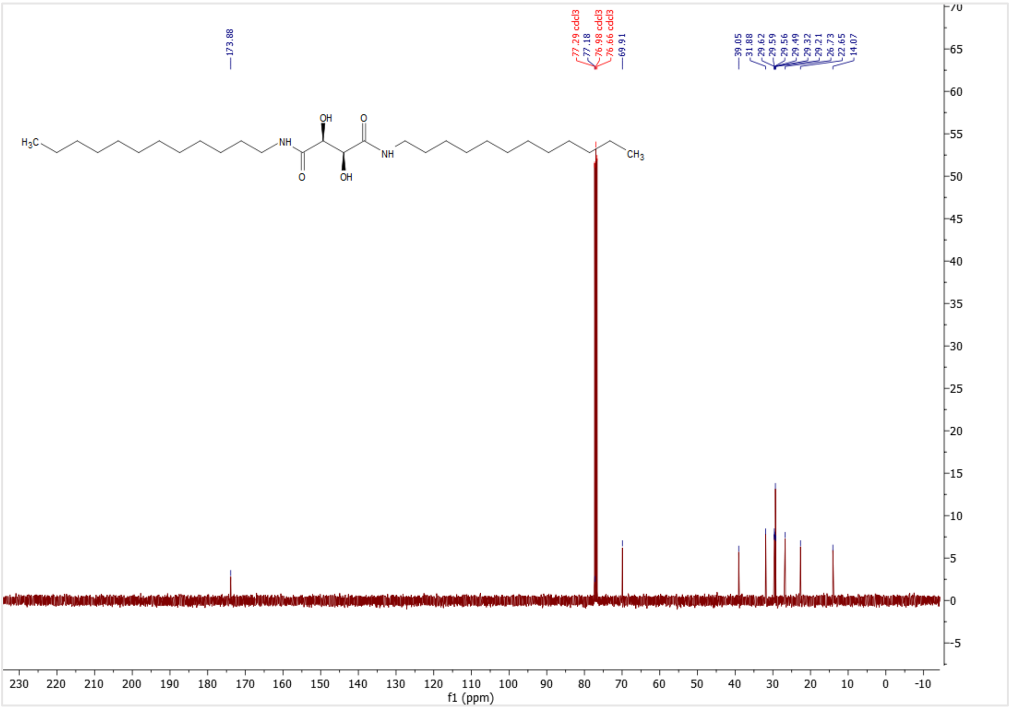

Figure S 8 ^{13}C NMR spectrum of D-(-)-tartaric acid didodecyl amide

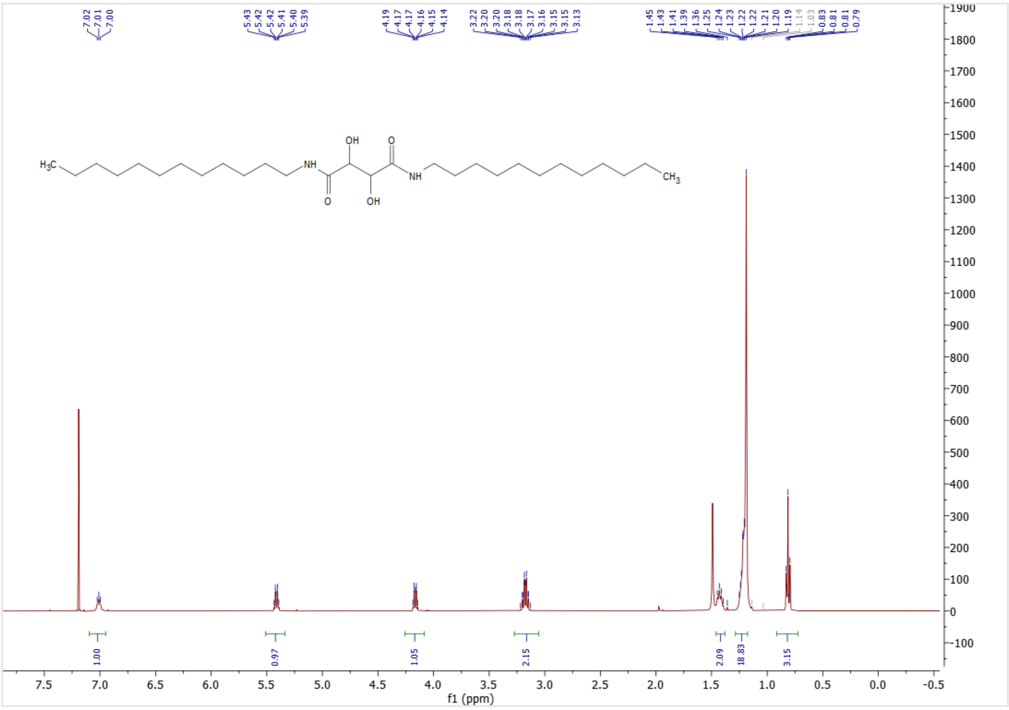

Figure S 9 ^{1}H NMR spectrum of DL-tartaric acid didodecyl amide

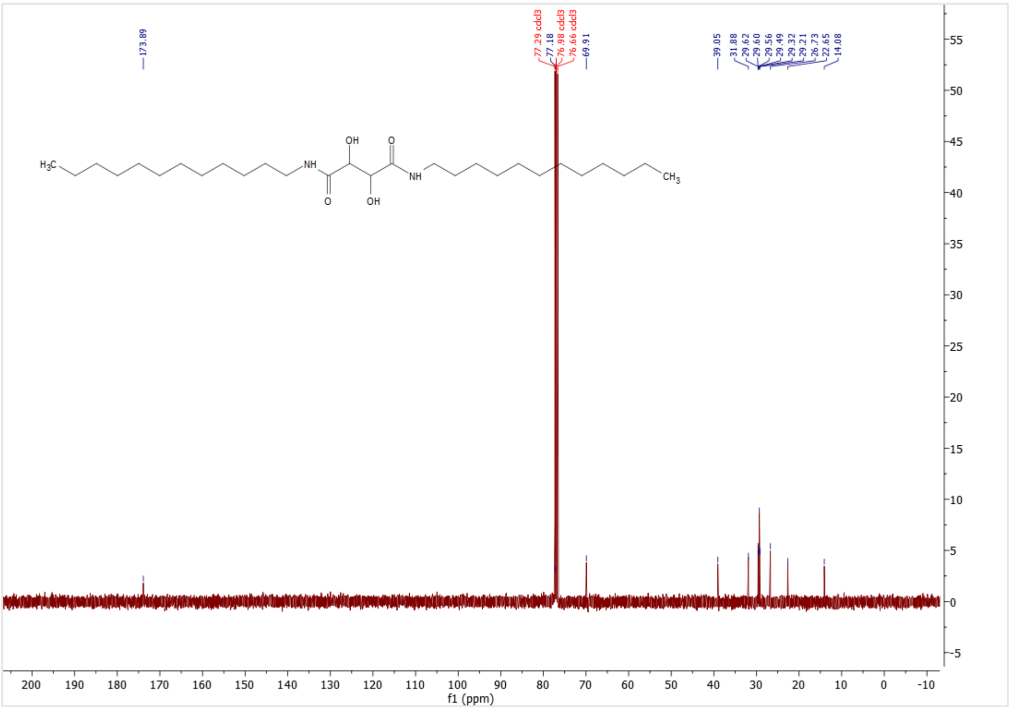

Figure S 10 ^{13}C NMR spectrum of DL-tartaric acid didodecyl amide

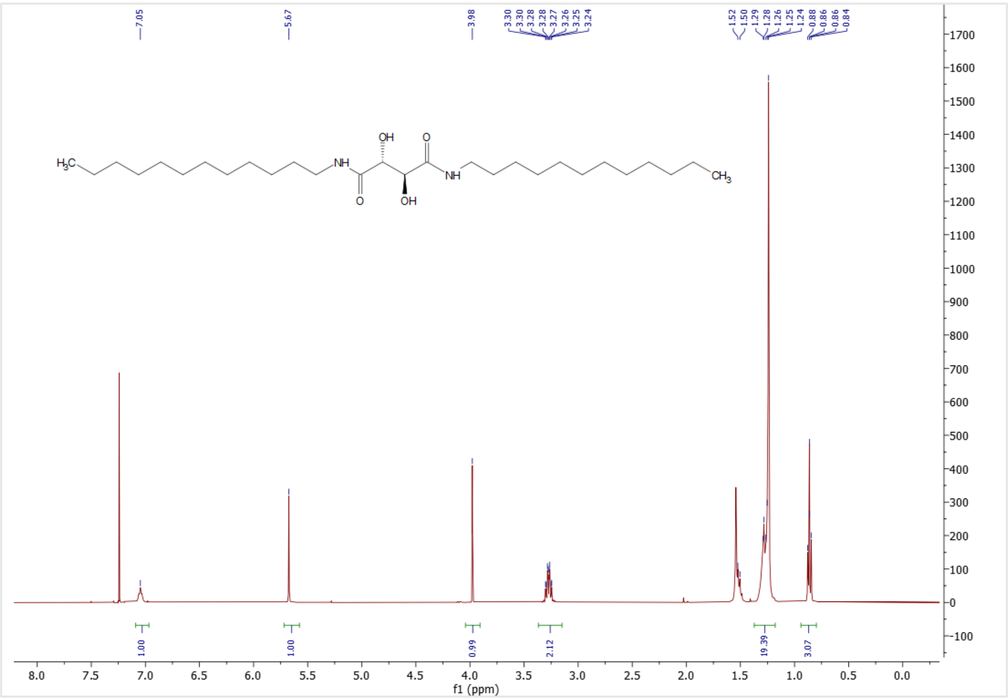

Figure S 11 ^{1}H NMR spectrum of meso-tartaric acid dodecyl amide

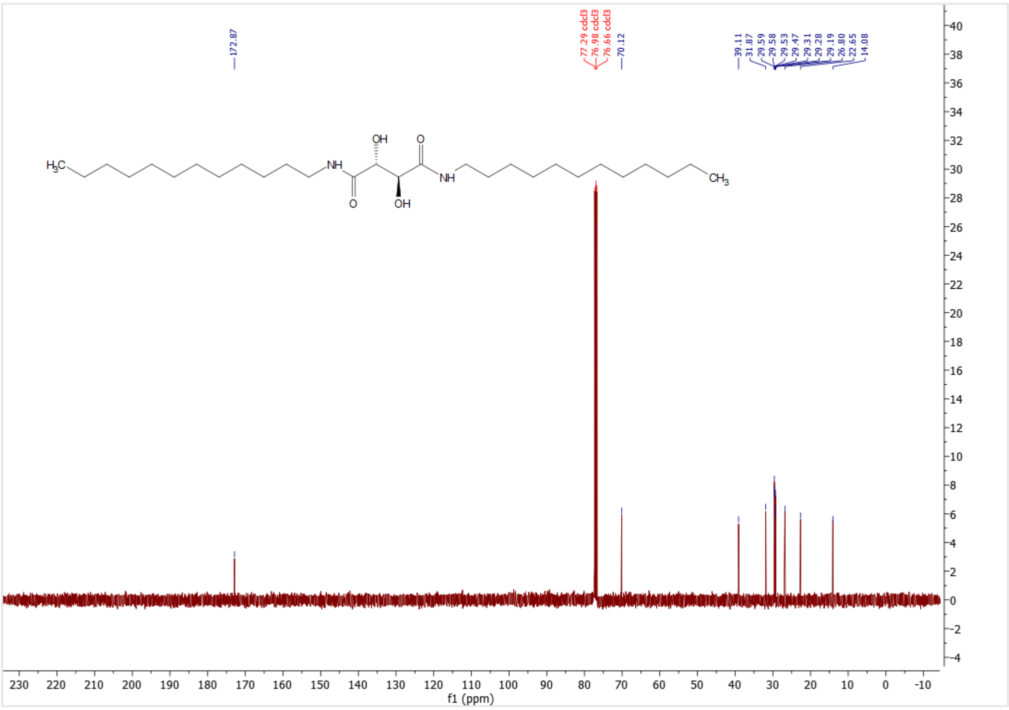

Figure S 12 ^{13}C NMR spectrum of meso-tartaric acid dodecyl amide

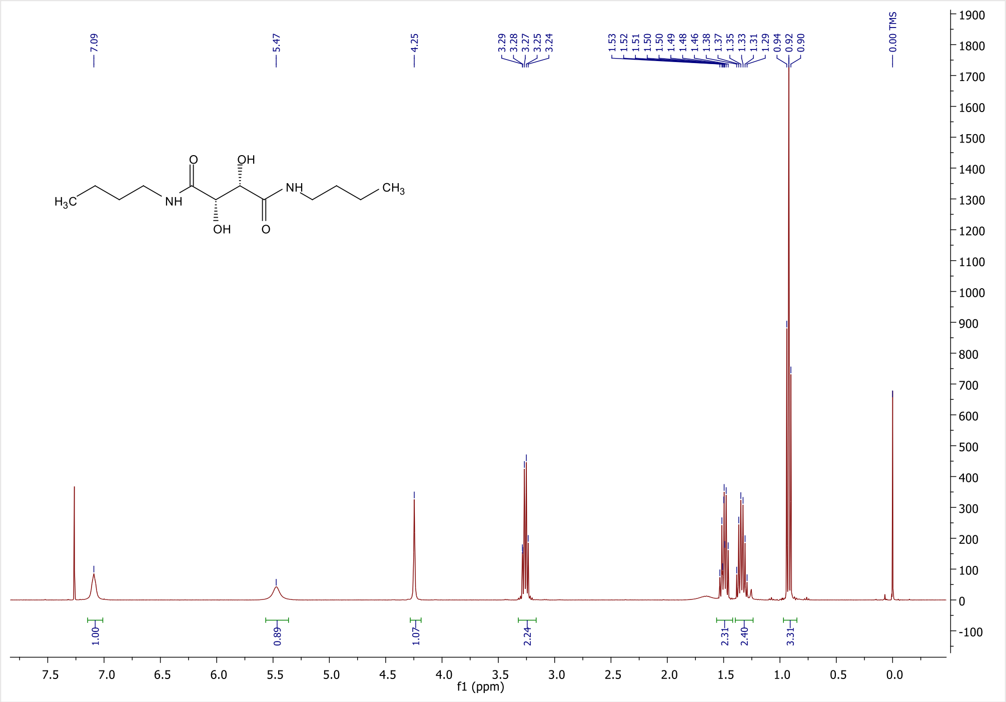

Figure S13 ^{1}H NMR spectrum of L-(+)-tartaric acid dibutyl amide

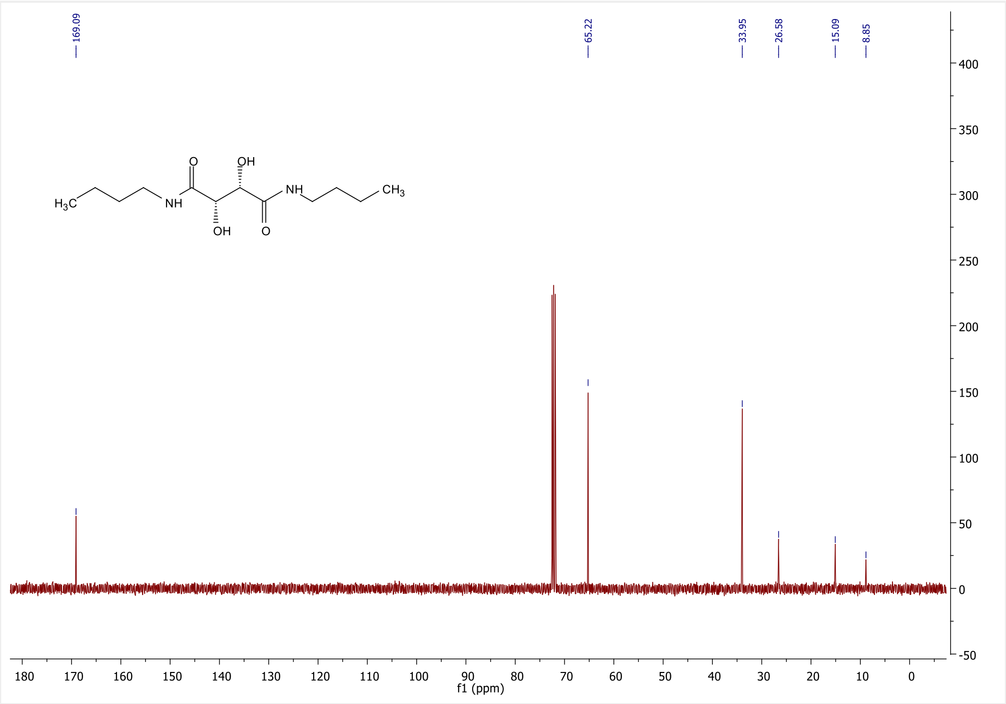

Figure S 14 ^{13}C NMR spectrum of L-(+)-tartaric acid dibutyl amide

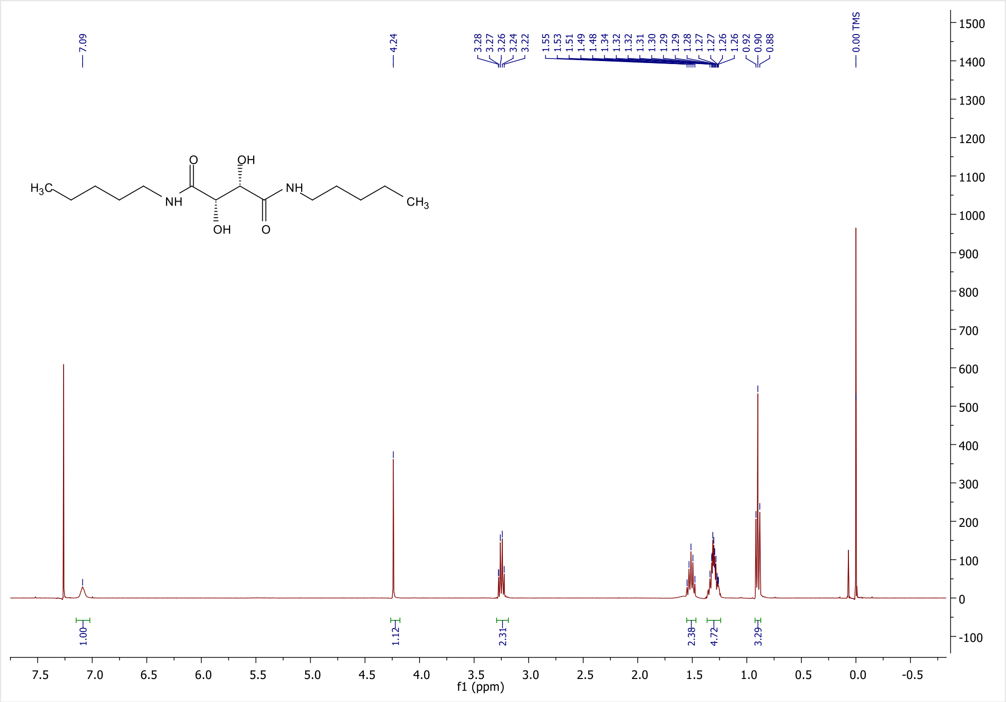

Figure S 15 ^{1}H NMR spectrum of L-(+)-tartaric acid dipentyl amide

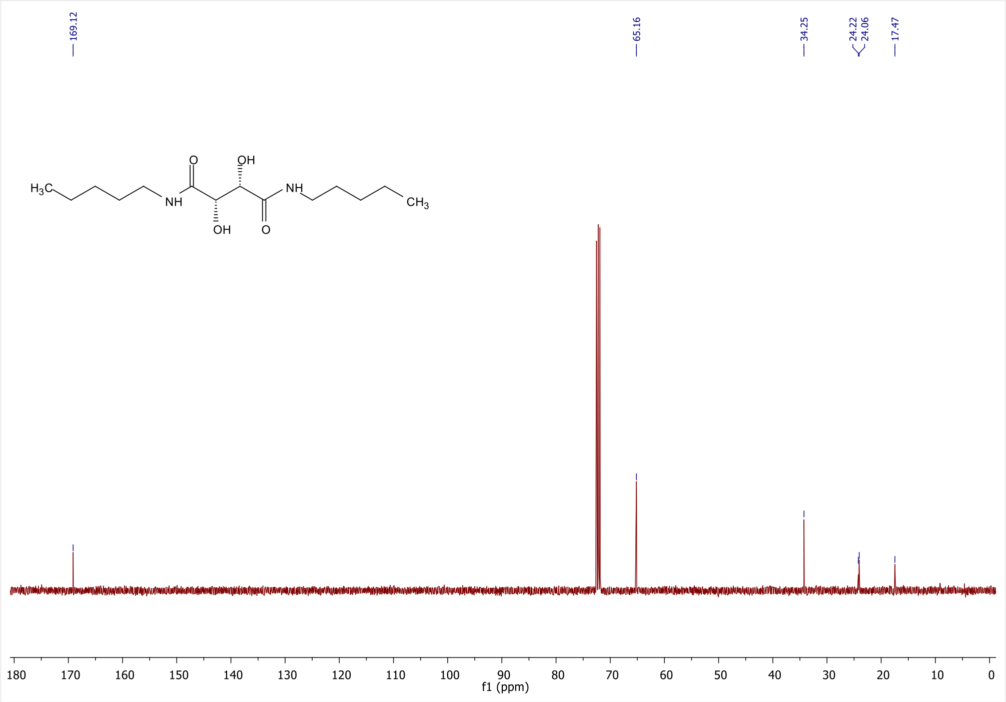

Figure S 16 ^{13}C NMR spectrum of L-(+)-tartaric acid dipentyl amide

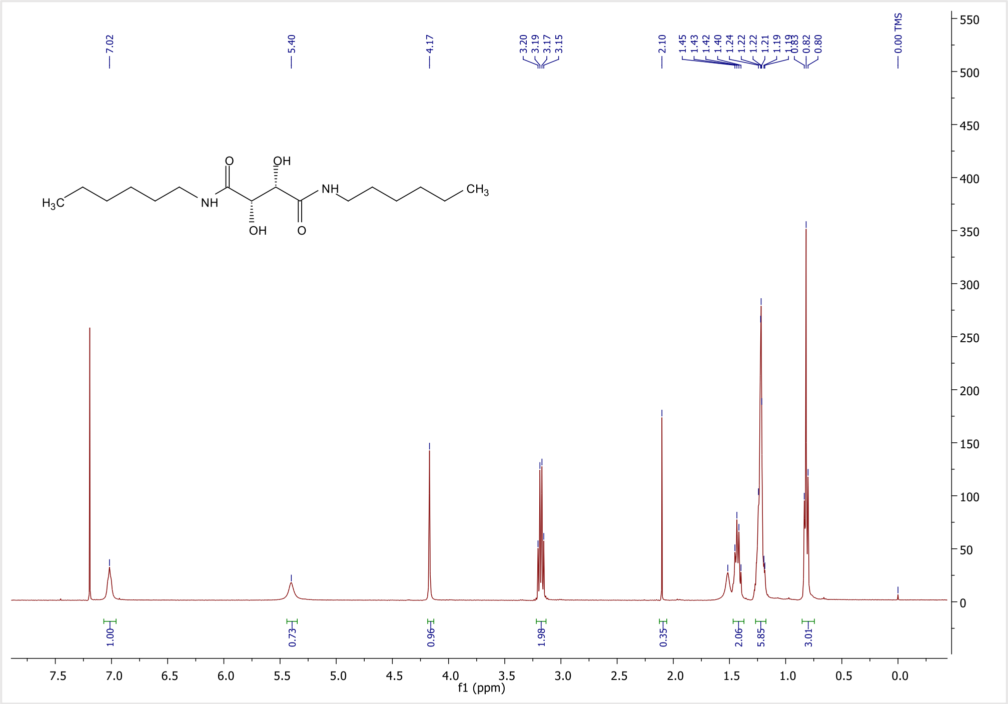

Figure S 17 ^{1}H NMR spectrum of L-(+)-tartaric acid dihexyl amide

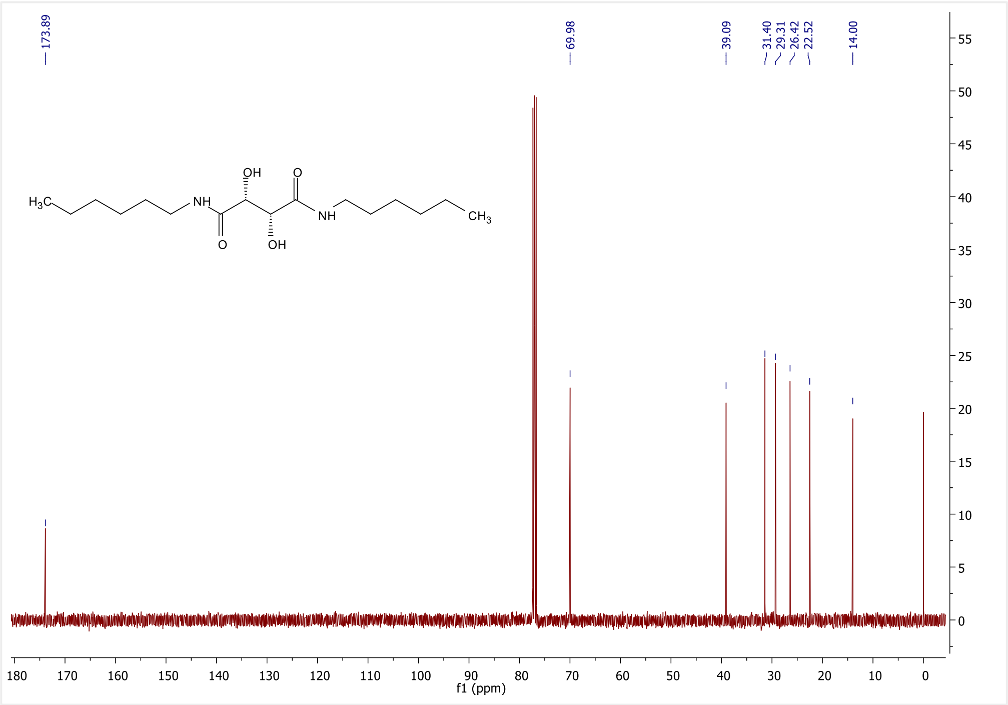

Figure S 18 ^{13}C NMR spectrum of L-(+)-tartaric acid Dihexyl amide

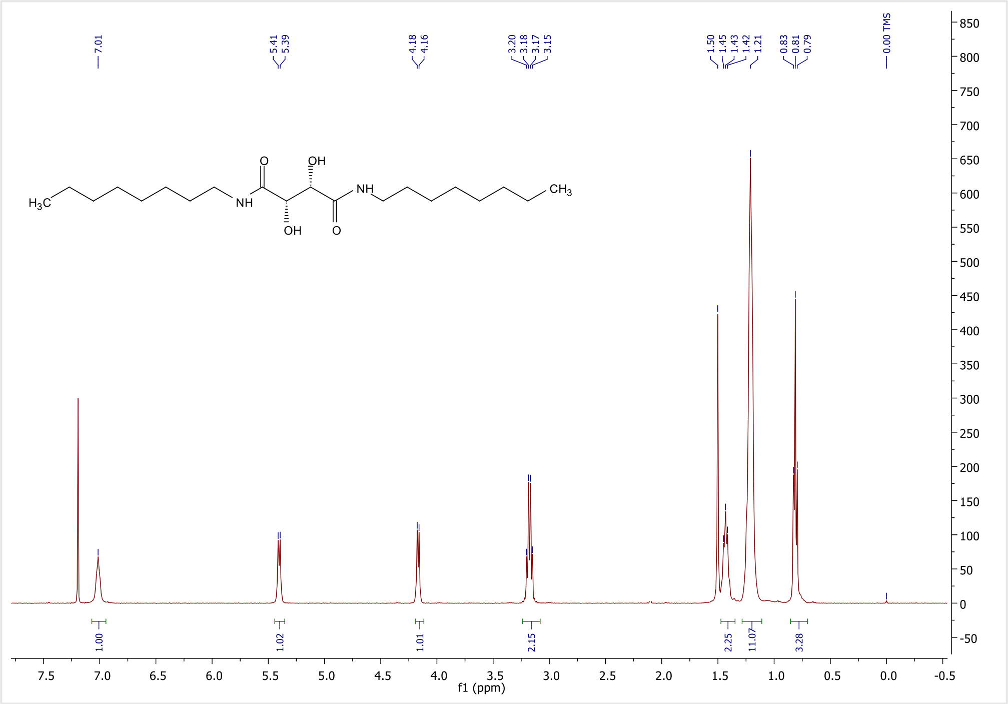

Figure S 19 ¹H NMR spectrum of L-(+)-tartaric acid dioctyl amide

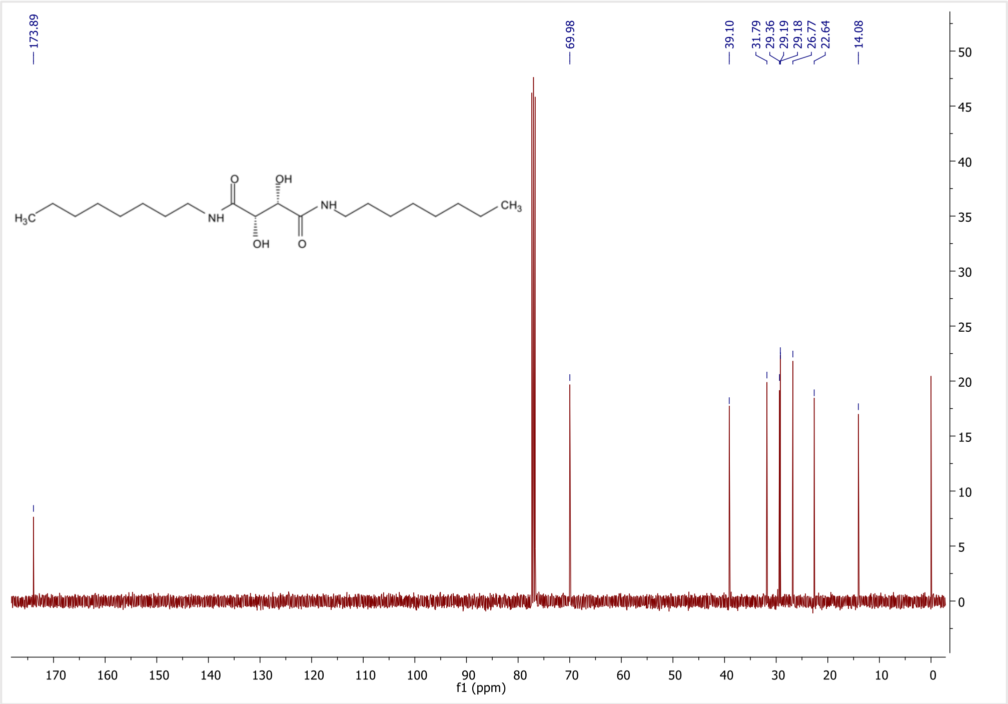

Figure S 20 ¹³C NMR spectrum of L-(+)-tartaric acid dioctyl amide

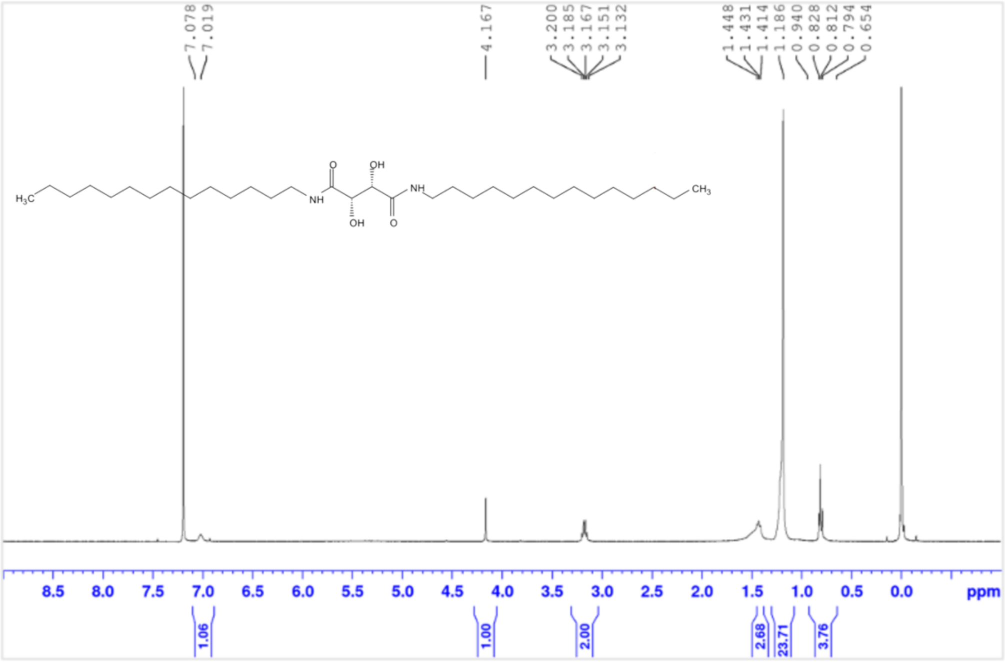

Figure S 21 ¹H NMR spectrum of L-(+)-tartaric acid ditetradecyl amide

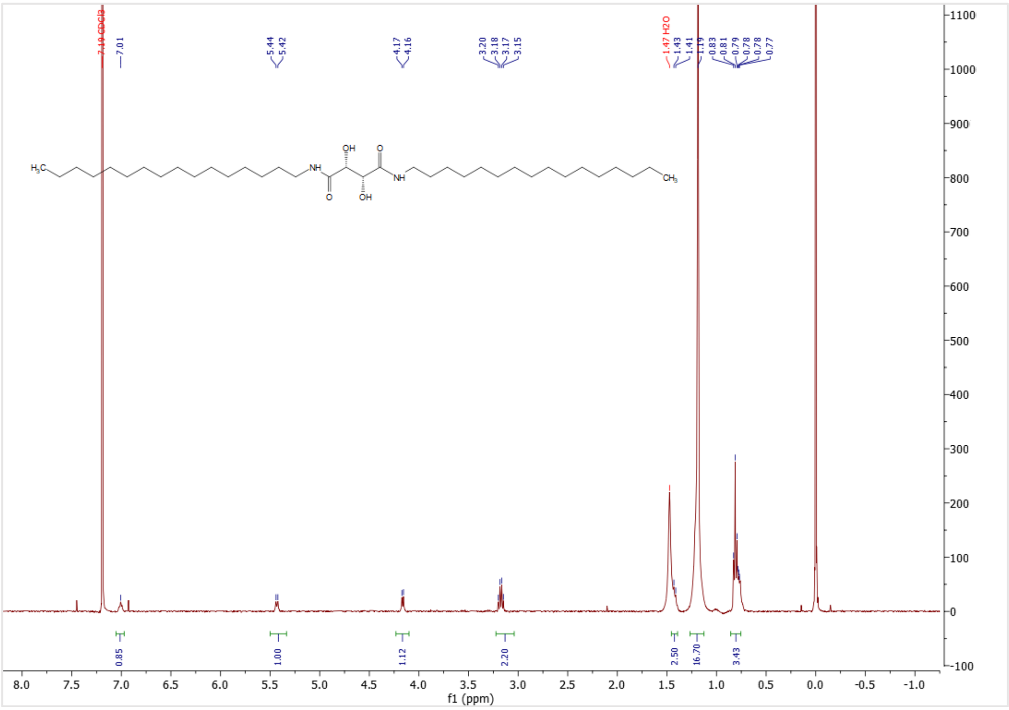

Figure S 22 1H NMR spectrum of L-(+)-tartaric acid dihexadecyl amide

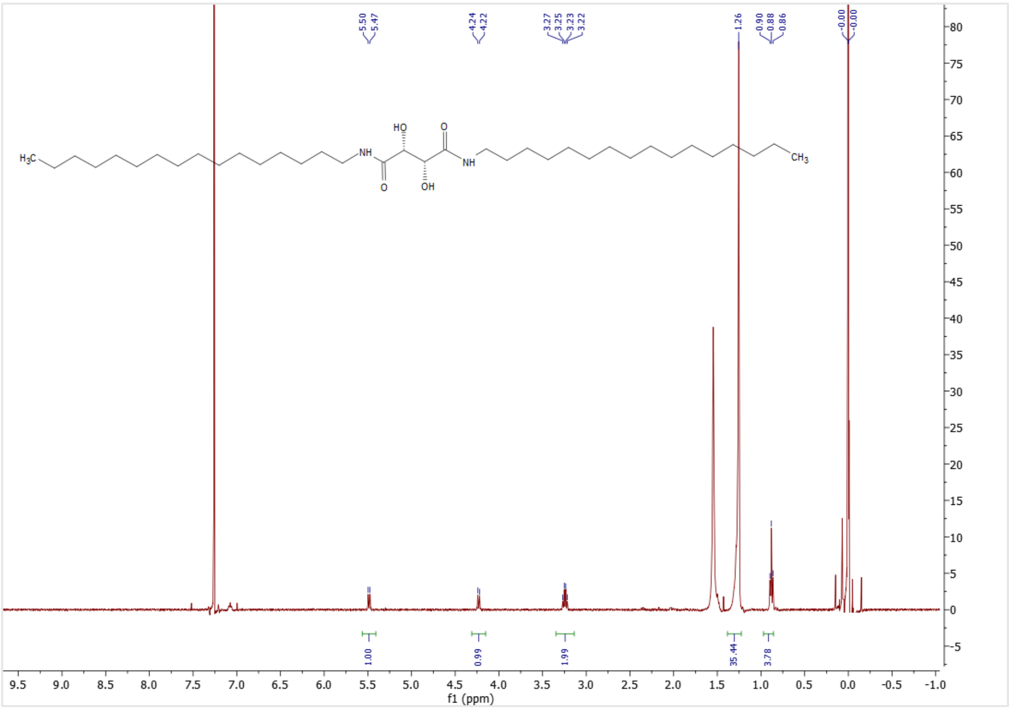

Figure S 23 ^{1}H NMR spectrum of L-(+)-tartaric acid dioctadecyl amide

FT-IR analysis

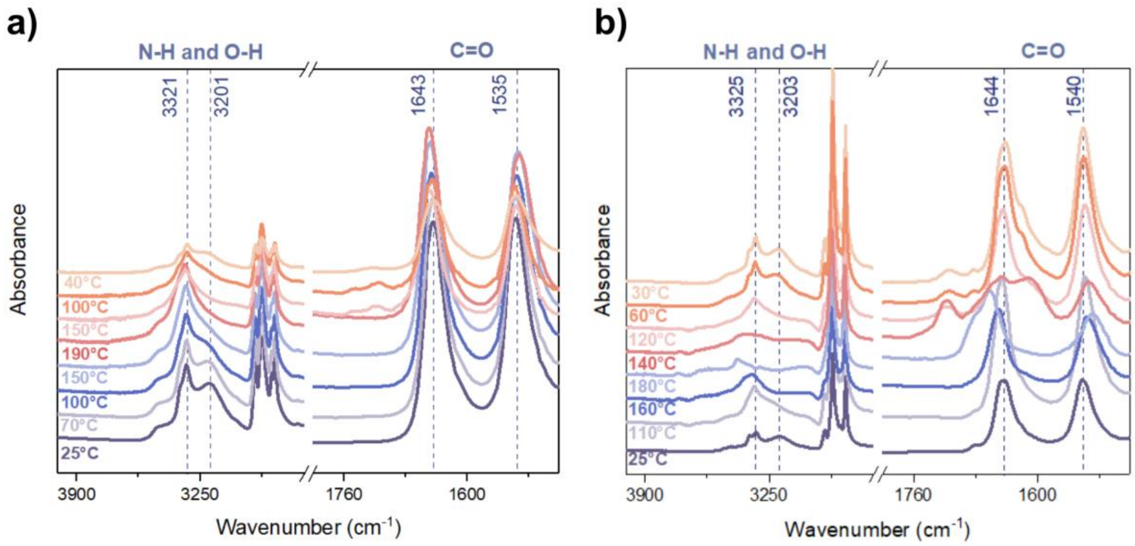

Figure S 24 FT-IR of L-(+)-DHA_6 and L-(+)-DDHA_16

TGA

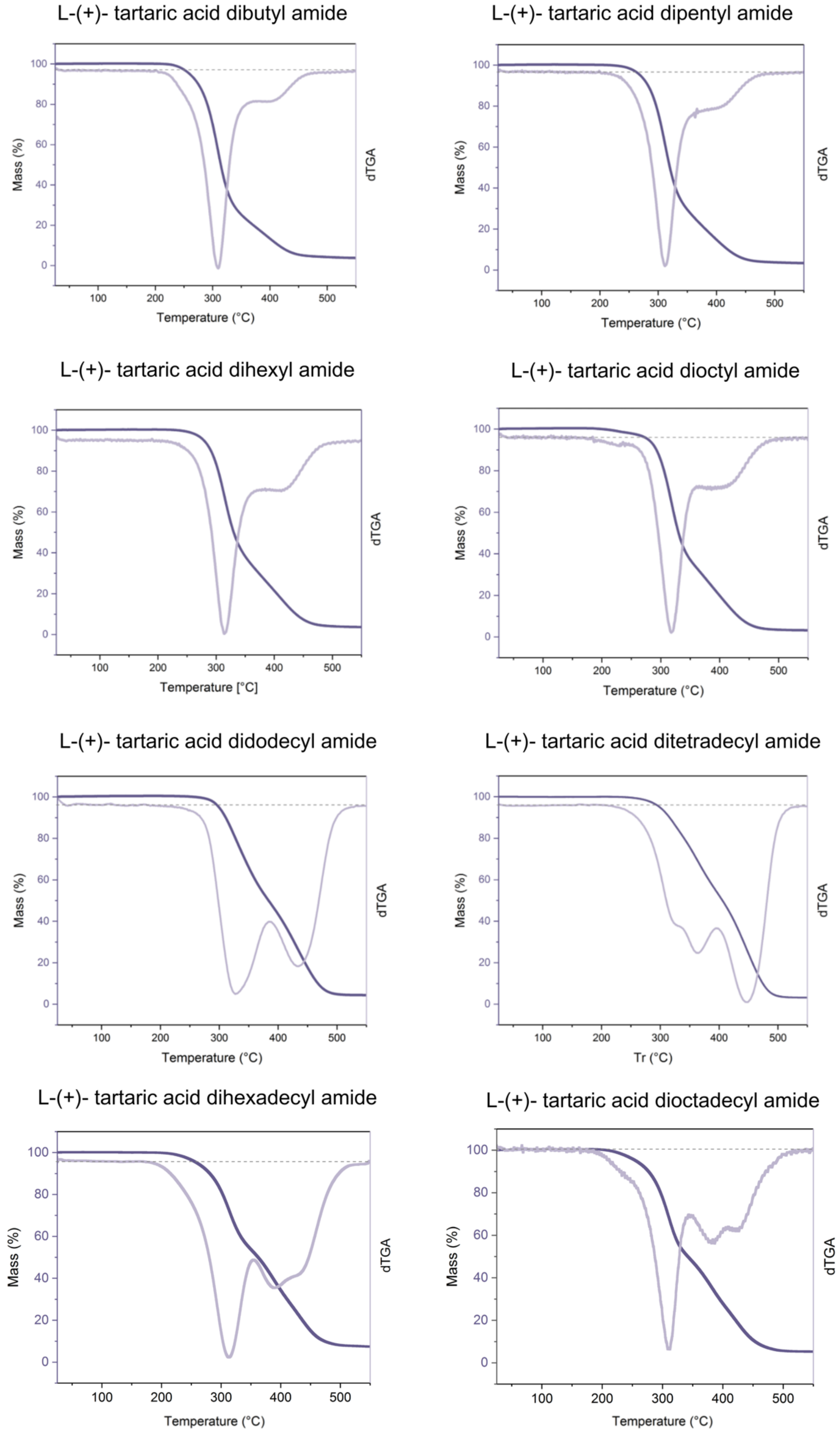

Figure S 25 TGA and DTGA of L-(+)-tartaric acid diamides

DSC

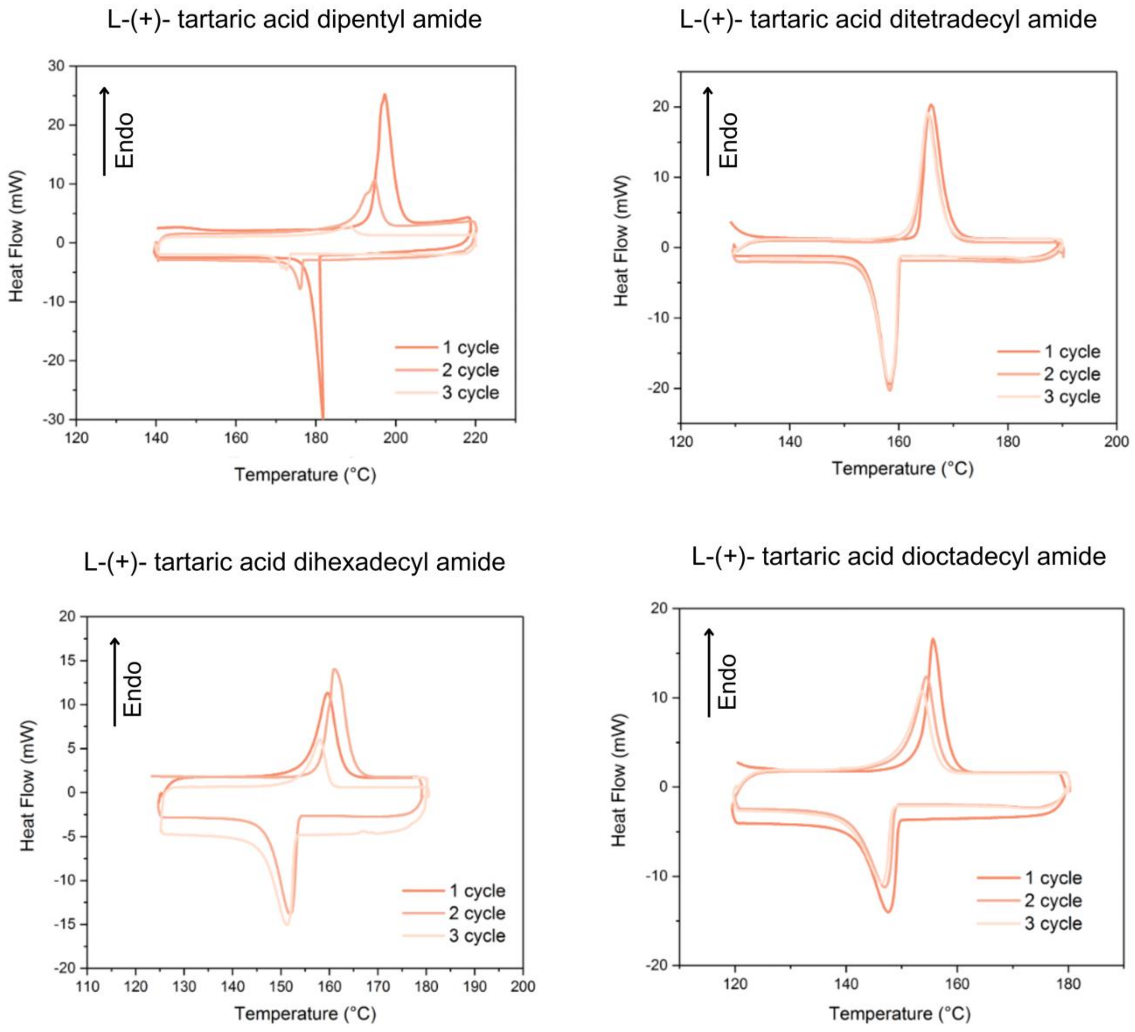

Figure S 26 DSC of L-(+)-tartaric acid diamides

Literature

[1] S. V. Gonzalez, P. Carlsen, *Arkivoc* **2011**, *2011*, 325.
